# What are the priorities of consumers and carers regarding measurement for evaluation in mental healthcare? Results from a Q-methodology study

**DOI:** 10.1186/s12961-024-01239-y

**Published:** 2024-11-11

**Authors:** Rachel O’Loughlin, Caroline Lambert, Gemma Olsen, Kate Thwaites, Keir Saltmarsh, Julie Anderson, Nancy Devlin, Harriet Hiscock, Kim Dalziel

**Affiliations:** 1 Health Economics Unit, School of Population and Global Health, University of Melbourne, Melbourne, VIC Australia; 2 Mental Health Improvement Program, Safer Care Victoria, Melbourne, VIC Australia; 3 Family and Carer Research, Tandem Carers, Abbotsford, VIC Australia; 4 School of Global, Urban and Social Studies, RMIT University, Melbourne, VIC Australia; 5 Health Services and Economics, Murdoch Children’s Research Institute, Parkville, VIC Australia; 6 Department of Paediatrics, University of Melbourne, Melbourne, VIC Australia; 7 Health Services Research Unit, The Royal Children’s Hospital, Parkville, VIC Australia

**Keywords:** Mental health, Mental health services, Q-sort, Patient reported outcome measures, Outcome assessment, Health care

## Abstract

**Background:**

The purpose of this study was to identify and describe common views of people with lived experience of mental health challenges – consumers and carers, families and supporters – of what they consider the most important measures to include in health economic evaluations which assess the incremental value of competing options in mental health care.

**Methods:**

Participants (*n* = 111) were people living in the state of Victoria, Australia, who identified as consumers of mental healthcare (*n* = 38); carers, family members and/or supporters (*n* = 43); or both (*n* = 30). Factor analysis based on Q-Methodology was used to identify clusters of people who hold similar viewpoints. Common viewpoints were described in terms of the characteristics of the group, and a qualitative interpretation was conducted on the basis of distinguishing statements and quotes provided in participants’ own words.

**Results:**

We identified four common views: (1) safety before all else, prioritizing physical, sexual and psychological safety; (2) hope and partnership in processes of care; (3) physical and emotional health and wellbeing; and (4) care access, continuity and partnership with families. Although different priorities were identified for each viewpoint, key priority areas that were common to all views were having an environment in the health service that fosters respect and dignity, and that consumers feel heard and listened to. In sub-group and qualitative analyses, differences were observed regarding the likelihood of consumers and carers holding each of the views, as well as by age group.

**Conclusions:**

While some differences were noted between the views of consumers and carers and different age groups, there was also common ground regarding what outcomes are of most importance to measure. Including these measures in evaluation frameworks would provide a way of focussing mental healthcare decisions on the aspects of mental healthcare that are of most value to consumers and carers, thereby addressing an important shortcoming of current approaches to decision-making in mental healthcare.

**Supplementary Information:**

The online version contains supplementary material available at 10.1186/s12961-024-01239-y.

## Highlights

- We identified four common viewpoints of consumers and carers in terms of what they consider the most important measures to include in health economic evaluations which asses the incremental value of competing options in mental health care: (1) safety before all else, prioritizing physical, sexual and psychological safety; (2) hope and partnership in processes of care; (3) physical and emotional health and wellbeing; and (4) care access, continuity and partnership with families.
- Key priority areas that were common to all views were having an environment in the health service that fosters respect and dignity, and that consumers feel heard and listened to. After these, each view was characterized by a different level of priority on additional areas of health and wellbeing, as well as processes and characteristics of care.
- Including the priority measures from each view within evaluation frameworks would start to incorporate the values and priorities of consumers and carers into mental healthcare decision-making, thereby addressing an important gap in current decision-making processes.

**Supplementary Information:**

The online version contains supplementary material available at 10.1186/s12961-024-01239-y.

## Introduction

The WHO has called for comprehensive action in mental healthcare [1] to address the increasing global burden of mental health challenges [2]. In Victoria, Australia, a recent Royal Commission into Victoria’s Mental Healthcare system [3] (hereafter Royal Commission) has initiated reforms within mental healthcare. However, a change in and of itself is not necessarily an improvement, and careful evaluation is required to know whether such changes are improving the lives of people with lived experience of mental health challenges, that is, service users, consumers, carers, families and supporters (hereafter, consumers and carers). Internationally, there is no clear consensus regarding how value should be defined and measured in healthcare [4]. In fact, how to define value is one of the top 10 issues impacting healthcare decision-making around the world [5]. Health economic evaluations conducted through national bodies such as the National Institute for Health and Care Excellence (NICE) in the United Kingdom, and the Medical Services and Pharmaceutical Benefits Advisory Committees (MSAC and PBAC) in Australia, traditionally assess competing interventions and treatments using a definition of value on the basis of “cost per quality-adjusted life year” (QALY) [6, 7]. In recent years, however, emerging value-assessment frameworks [8] are encompassing a wider set of outcomes, impacts and equity considerations. Arguments have also been made [9] that health economic analyses should assess the impact of healthcare interventions across a range of measures aligned with the values and priorities of society. In the United Kingdom, for example, there has been a push for healthcare to not only be value-based, but “values-based”, by focussing on patients’ wellbeing, quality of life, independence, social connectedness, choices and equity [10, 11]. Healthcare policy decision-making based on measures of this type, in contrast to more narrowly defined costs and QALYs, may result in different conclusions about the value of competing interventions. The way in which value is defined therefore becomes critical to ensuring the most appropriate measures are captured and utilized to inform healthcare policy financing and decision-making.

Increasingly, health systems are embracing the knowledge of consumers and carers as experts in their own care [1, 3], with calls for the planning, delivery and evaluation of mental healthcare to be conducted in partnership with people with lived experience [12, 13]. Specifically, a major recommendation arising from the Royal Commission was the need to hear and partner with people with lived and living experience of mental health challenges [3], and the Victorian state government is endeavouring to address this recommendation [14]. Internationally, the WHO, as well as the national governments of the United Kingdom, Australia and Canada, recommend consumers and carers be involved in decision at all levels of healthcare policy, research and decision-making [1, 15–17]. This speaks to an increasing desire for the voices of consumers and carers to be reflected and empowered within mental healthcare and policy decision-making. While this is becoming more commonplace, the level of engagement has been described as tokenistic [13]. Moreover, a recent systematic review [18] noted that only 11 of 57 value assessment frameworks were developed with any level of consultation with people with lived experience, and none of the frameworks were developed specifically for use in mental healthcare. These findings reveal that the voices of consumers and carers are not yet genuinely reflected in value assessment in mental healthcare [18], yet there is a strong desire for this to be achieved in healthcare policy decision-making.

As a recent exception to this, a qualitative study with adult mental health service users (*n* = 14) and mental health professionals (*n* = 8) in the United Kingdom [19] found six key themes to be important in assessing the quality of mental healthcare: accessing care; assessing the benefits of care; coordinated care; delivery of mental healthcare; individualized care; and the role of the person providing care. This study, however, did not include the views of carers. This is notable, as studies aiming to elicit research area priorities in mental healthcare [20, 21] have shown that the views of consumers and carers can diverge substantially in some areas, with both groups substantially impacted. It is possible that the views of consumers and carers similarly diverge when examining priorities for value assessment in mental healthcare, however, this has not been previously explored. It would be important to explore these views separately, as it is recognized that consumers and carers have different needs, experiences and perspectives, which has led to recommendations to explicitly engage with both consumers and carers in all areas of mental healthcare including, for example, mental health training standards [22], clinical care and relationships [23] and service improvement and evaluation efforts. [3]

Further to the difficulty in defining value in mental healthcare – in general – definitions and measures of value may differ for child versus adult mental healthcare. The differences in the structure of healthcare for children and young people, and the relative prominence of prevention, mean that conceptual debates regarding the value of mental healthcare should consider value in young people’s healthcare independently of adult care, yet there is far less literature and consensus on these concepts in paediatric care [24]. Equally, the views of older adults, what is important to them, how they view their mental healthcare, and the unique complexities for mental healthcare in this cohort have been shown to substantially differ from other age groups [25]. However, to our knowledge, there is no published literature that specifically describes the views of children and young people, or older adults, regarding what outcomes should be measured for value assessment in mental healthcare.

Arising from these significant gaps in existing knowledge, we aimed to identify and describe the common viewpoints of consumers and carers in terms of what they consider the most important measures to include in health economic evaluations of mental healthcare. Specifically, we aimed to (1) identify the most common viewpoints/priorities of consumers and carers and (2) describe the similarities and differences in these viewpoints in relation to group characteristics (including consumers, carers, young people, adults and older adults).

## Methods

### Study design

This study was a cross-sectional, online survey structured and analysed using Q-methodology [26]. Q-methodology combines qualitative and quantitative methods to identify and compare the subjective viewpoints of individuals or groups by identifying clusters of people who hold similar viewpoints on a topic [26]. For this reason, Q-methodology has been used previously to study the priorities of different stakeholder groups to inform priority-setting in general healthcare [27–29] and mental healthcare [29]. The processes of statement development, data collection and analysis and reporting for this study were conducted in line with published guidelines for Q-methodology studies. [26, 28, 29]

The study was a partnership between the University of Melbourne, Safer Care Victoria (Victorian Government) and two peak bodies for consumers and carers in Victoria, Australia: Tandem Carers and the Victorian Mental Illness Awareness Council (VMIAC). The study received ethics approval from the University of Melbourne Human Research Ethics Committee (ref. 2023-24566-36658-6), and all participants provided informed consent prior to completing the survey.

### Developing the ranking task: the Q-set

In Q-methodology, participants respond to a set of statements called the Q-set. The aim is to have a balanced Q-set that encompasses a broad range of potential priority areas, allowing all participants to express their views without feeling limited or restricted [26]. An initial Q-set was developed by the research team through a combination of literature review; items from existing instruments designed to measure service user perceptions of, satisfaction with and outcomes of mental healthcare; and 1:1 meetings with professionals in the fields of health economics, mental health research, policy and mental health clinical care. This initial phase of “sampling the concourse” [29] resulted in a preliminary Q-set of 27 statements. The Q-set was designed to include a range of outcomes, process measures and aspects of service quality and delivery to explore the priorities of consumers and carers around the delivery, experience and outcomes of mental healthcare. This initial Q-set was subsequently workshopped with a Lived Experience Advisory Group comprising eight people with lived experience of mental health challenges, including consumers and carers, from across the partner organizations for the study. Workshopping took place over approximately 3 months until all lived experience advisors felt the language, content, and breadth of the Q-set appropriately captured the range of potential responses and was evenly balanced for the views of consumers and carers.

### Piloting and the final Q-set

Following initial development and workshopping, a draft Q-set of 41 statements, with response options on a 7-point scale, was initially piloted with the Lived Experience Advisory group. This format was found to be too complex to complete and resulted in two major changes to the Q-set and response format.

First, in collaboration with the Lived Experience Advisory group, the Q-set was reduced to a final total of 30 statements (Table 1), which falls within the accepted range of 25–80 statements [26]. Given that we were required to include fewer statements for feasibility within the target cohort, we used the approach outlined by Watts and Stenner [26] of including broader statements to ensure the Q-set remained representative of the possible range of viewpoints.

**Table 1:** Full set of statements: the Q-set

*Q-set shown in online survey*	*Abbreviation used in text*
1. Consumers’ experience of mental health symptoms	*(#1) mental health symptoms*
2. Consumers’ and family/carer/supporters’ sense of hope and optimism	*(#2) hope and optimism of consumer/carer*
3. Consumers’ sense of identity and meaning	*(#3) identity and meaning*
4. The family/carer/supporter’s overall wellbeing	*(#4) carer wellbeing*
5. Consumers’ physical health	*(#5) physical health*
6. Consumers’ ability to conduct daily tasks	*(#6) conduct daily tasks*
7. The sense of physical and sexual safety in the healthcare setting	*(#7) physical and sexual safety*
8. The sense of psychological safety in the healthcare setting and in interactions with staff	*(#8) psychological safety*
9. The personal and economic impacts of accessing care (e.g. safe housing, relationship, jobs), for consumers, families/carers/supporters	*(#9) personal cost*
10. Consumers’ relationships with those who are important to them as well as others	*(#10) interpersonal relationships*
11. An environment in the health service that fosters respect and dignity	*(#11) respect and dignity*
12. The level of respect and understanding shown for personal values that are important to consumers and families/carers/supporters, including connection to culture, faith based and/or spiritual values and gender identity	*(#12) respect for values*
13. A culture of hope and optimism in the care provided by the clinician(s) and professional staff	*(#13) hope and optimism of clinicians*
14. The way that families/carers/supporters are supported by the health service	*(#14) support for carers*
15. Access to healing activities, spaces and places	*(#15) healing activities and spaces*
16. Access to peer support throughout the journey with mental health services	*(#16) peer support*
17. How supported consumers feel to continue their recovery journey	*(#17) supported through journey*
18. The continuity of care experienced	*(#18) continuity of care*
19. The level of access to the treating doctor or psychiatrist when needed	*(#19) access to doctor*
20. Whether consumers feel listened to and feel heard	*(#20) consumers feel heard*
21. A sense of partnership and listening to families/carers/supporters throughout the journey with mental health services	*(#21) partnership with families*
22. The quality and timing of information provided to families/carers/supporters about their role and the ongoing recovery of the consumer	*(#22) quality and timing of info*
23. Whether consumers’ and families/carers/supporters’ human rights are upheld	*(#23) human rights*
24. Families and consumers experience discharge that is informed, supported and sustainable	*(#24) safe discharge*
25. Consumers’ privacy and physical comfort in the service	*(#25) privacy and comfort*
26. Whether (paid) care teams have an appropriate mix of skills and capabilities for consumers’ needs	*(#26) skilled teams*
27. Whether the service provides better access for those who need it most	*(#27) equitable access*
28. Whether the service provides more equal (or equitable) outcomes for people accessing care	*(#28) equitable outcomes*
29. The convenience and accessibility of the location of the service	*(#29) convenience of service*
30. The cost: the amount of money consumers, and families/ carers/supporters have to pay for care	*(#30) cost of care*

NB: The left hand column of this table shows each statement as it was presented in the online survey. On screen, each statement was followed by a “(?)” symbol. When participants hovered over this symbol, they were provided with additional information on each item. The full description of each item can be found within the full online survey available in Supplementary Material 1. The right hand column of this table shows the abbreviated items referred to in the manuscript

Second, response options were simplified from a 7-point to a 5-point scale, ranging from −2, least important; to 0, neutral; to +2, most important. This scale was used to construct the final Q-sort response grid (Supplementary Figure S2.1), following the standard approximately normal shape [26], where a set number of items are categorized at each level.

### Sample size, participants and recruitment

Q-methodology studies generally have smaller sample sizes, similar to those found in qualitative studies. The aim for sampling in Q-methodology studies is to include as many individuals as required to establish the existence of different viewpoints, and is not to determine what proportion of the population hold that view [26]. Therefore, there is no recommended target sample size, though a ratio of one participant for every two items in the Q-set has been suggested as a guide [26]. With a final Q-set of 30 statements, a sample size of approximately 15 participants in each sub-group of interest (i.e. consumer/carer and age groups) was considered sufficient.

Participants were purposively sampled for people with lived experience of mental health challenges, with an approximately equal mix of consumers, carers, and those who identify as both a consumer and carer. Participants were recruited via email newsletters distributed through networks associated with the two partner organizations, Tandem Carers and VMIAC, and were reimbursed for their time completing the online survey. All participants were required to be at least 15 years old and have sufficient English to provide consent and understand and complete the task.

### Data collection procedures—“Q-sorting”

Participants completed the survey online, individually, or with the help of a support person if desired, using the online survey tool Qualtrics. The full survey is available in Supplementary Material 1, and included demographics questions, short instructional videos, the Q-sort task and a post-sorting section that allowed participants to describe the strongly-ranked statements in their own words, as well as provide any other general comments.

The Q-sort task was structured in line with Watts and Stenner’s provisional ranking categories process [26]. This means participants were presented with the full 30-item Q-set and asked to first indicate the 10 items they felt most strongly were important to measure and the 10 items they felt most strongly were not important to measure. From each of these 10-item lists, participants were then asked to choose the 3 items they felt the very most and very least important to measure. From these decisions, we were able to construct the Q-sort distribution grid for each participant (Supplementary Figure S2.1), rather than participants sorting the statements directly into this grid. This response format was chosen through the piloting phase in response to feedback from the Lived Experience Advisory group, who felt the direct sorting method added unnecessary complexity to the task, where they felt they were simply trying to “complete” the task correctly, and this took attention away from considering the items carefully for their own merit.

At any stage of the ranking process participants were able to return to a previous step and adjust their responses.

### Statistical analysis

#### Quantitative factor analysis

Q-methodology uses a type of “by-person” factor analysis [26] where each variable represents one person’s viewpoint. Each factor therefore represents a group of individuals who hold a similar viewpoint or set of preferences. Factor analyses were conducted using the qfactor program [30] in StataSE 16 (Statacorp, Texas, United States). Principal component analysis was used to examine the patterns and themes emerging from the data. The number of principal components (hereafter factors) to be retained was determined on the basis of a combination of three commonly used methods [31]: (1) the Kaiser–Guttman criterion of eigenvalues greater than 1.00; (2) examination of the scree plot; and (3) Horn’s Parallel analysis, using 3330 iterations of random data. In addition, we accepted only factors that had at least two significantly loading Q-sorts [26]. Factors were rotated using the orthogonal Varimax method to allow for greater interpretability of each factor.

Factor scores were calculated using Brown’s method [32], and represent the correlation between each participant’s Q-sort and each factor. Participants were loaded onto a factor (i.e. considered part of this group) if their factor score was statistically significant (*p* < 0.05). Distinguishing statements (those that are rated significantly differently for each factor), and consensus statements (those that are rated similarly for all factors), were identified firstly using Stephenson’s method [33]. However, as the distinguishing and consensus statements are relied heavily upon to guide the interpretation of each viewpoint, we conducted a secondary sensitivity analysis using a higher threshold for significance for determining these distinguishing and consensus statements, using Cohen’s effect size > 0.80. [34]

##### Sub-group analyses

To examine views within our sub-groups of interest, we conducted a series of analyses that repeated the factor analysis process within separate sub-groups of consumers; carers; and those who identify as both a consumer and a carer (hereafter consumer + carers); as well as separately by age group (young people aged 15–24 years; adults aged 25–64 years; and older people aged 65+ years). The aim of this series of analyses was to identify common viewpoints that emerge when considering each sub-group separately, and to then explore whether these viewpoints were being accurately captured and represented within the main analysis. Crosstabs were used to examine the overlap of views within these sub-groups with the views emerging from the main (total sample) analysis.

##### Qualitative interpretation of viewpoints

Following quantitative analyses, we conducted a qualitative interpretation of the viewpoints. A factor array was constructed for each view [26], which is a single Q-sort that represents an average of the individual rankings within each view. These factor arrays, and distinguishing and consensus statements, were drawn upon to interpret each viewpoint in line with the crib sheet method of Watts and Stenner [26]. This means the interpretation of each viewpoint considers not only the items each group consider very important to measure, but those that are considered less important, or for which they had no strong feelings, with similarities and differences described relative to other views. Indicative quotes in participants’ own words are presented to describe the key concepts that characterize each viewpoint and aid in the qualitative interpretation of each viewpoint. Differences in group characteristics for each factor were explored qualitatively.

## Results

### Sample characteristics

A total of 111 participants completed the online survey and were included in the analysis. Sample characteristics for the total sample and across sub-groups of interest are detailed in Table 2. While a range of demographics were sampled, many participants were female (78.3%); identified as Australian (64.8%; though many of these participants also reported a range of secondary cultures); were primarily English speaking (93.6%); and lived in a major city of Australia (78.3%). Most consumers had been experiencing mental health challenges for more than 10 years (65.7%); had accessed services for their mental health within the last year (84.2%); and some (42.1%) had an overnight stay in hospital for mental health reasons in the last 5 years. Carers were supporting people across the full spread of ages (from 0 to 14 years to 65+ years); the majority had been supporting this person/people for more than 10 years (55.8%); and the person/people they cared for had accessed services within the last year (90.7%); or been admitted for an overnight stay in hospital within the last 5 years (62.7%). Compared with consumers, fewer consumer + carers had been experiencing their own mental health challenges for more than 10 years (53.3%), or had an overnight stay in hospital in the last 5 years (26.6%). Compared with carers, fewer consumer + carers had been supporting the person/people they cared for for more than 10 years (33.3%), and fewer people they cared for had been admitted for an overnight stay in hospital within the last 5 years (36.6%).

**Table 2:** Sample characteristics for the total sample and four most common viewpoints

Characteristics	Total sample *n* (%)	Common viewpoints			
		Viewpoint 1 *n* (%)	Viewpoint 2 *n* (%)	Viewpoint 3 *n* (%)	Viewpoint 4 *n* (%)
Total completed surveys	111	23	21	16	15
*Mental health lived experience*					
Consumer, only	38 (34.2)	9 (39.1)	4 (19.0)	9 (56.2)	4 (26.6)
Family member, carer, supporter, only	43 (38.8)	5 (21.7)	9 (42.8)	5 (31.2)	9 (60.0)
Both consumer and family/carer/supporter	30 (27.0)	9 (39.1)	8 (38.1)	2 (12.5)	2 (13.3)
*Age*					
15–24 years	16 (14.4)	3 (13.0)	5 (23.8)	1 (6.2)	1 (6.6)
25–64 years	82 (73.8)	18 (78.2)	16 (76.2)	15 (93.8)	12 (80.0)
65+ years	13 (11.7)	2 (8.7)	0 (0.0)	0 (0.0)	2 (13.3)
*Gender*					
Female	87 (78.3)	22 (95.6)	16 (76.1)	12 (75.0)	12 (80.0)
Male	21 (18.9)	1 (4.3)	3 (14.2)	4 (25.0)	3 (20.0)
Non-binary	2 (1.8)	0 (1.8)	2 (9.5)	0 (0.0)	0 (1.8)
*Prefer not to say*	1 (0.9)	0 (0.9)	0 (0.0)	0 (0.0)	0 (0.9)
*Cultural and ethnic background (primary stated)*					
Australian*	72 (64.8)	15 (65.2)	15 (71.4)	12 (75.0)	8 (53.3)
English	16 (14.4)	7 (30.4)	1 (4.7)	1 (6.2)	3 (20.0)
Other European (*including Afghan, Bosnian, Hungarian,* *Irish, Russian, Scottish*)	10 (9.0)	0 (0)	2 (9.5)	2 (12.5)	1 (6.6)
Asian (*including Chinese, Filipino, Indian, Korean,* *Nepalese*)	8 (7.2)	1 (4.3)	2 (9.5)	1 (6.2)	21 (13.3)
North American (*including American, Canadian*)	2 (1.8)	0 (0)	1 (4.7)	0 (0)	1 (6.6)
African (*including Kenyan, Mauritian*)	2 (1.8)	0 (0)	0 (0)	0 (0)	0 (0)
*Not stated*	1 (0.9)	0 (0)	0 (0)	0 (0)	0 (0)
*Primary language spoken at home*					
English	104 (93.6)	22 (95.6)	19 (90.4)	16 (100)	14 (93.3)
Other Language (*Stated as: Mandarin, Nepali,* *Hungarian, Hebrew, Cantonese, Swahili, Dari*)	7 (6.3)	1 (4.3)	2 (9.5)	0 (0)	1 (6.6)
*Aboriginal and/or Torres Strait Islander*					
Non-indigenous	104 (93.6)	22 (95.6)	17 (80.9)	15 (93.7)	15 (100)
Aboriginal†	3 (2.8)	0 (0)	3 (14.2)	0 (0)	0 (0)
*Prefer not to say*	4 (3.6)	1 (4.3)	1 (4.7)	1 (6.2)	0 (0)
*Highest level of education*					
Bachelor or above	48 (43.2)	10 (43.4)	8 (38.1)	6 (37.5)	8 (53.3)
Certificate I/II/III/IV (including trade certificate)	30 (27.0)	6 (26.0)	7 (33.3)	7 (43.7)	4 (26.6)
Completed year 12 (or equivalent)	18 (16.2)	5 (21.7)	3 (14.2)	2 (12.5)	1 (6.6)
Some school, but not completed year 12 (or equivalent)	14 (12.6)	2 (8.7)	3 (14.2)	1 (6.2)	2 (13.3)
Still at school (in high school)	1 (0.9)	0 (0)	0 (0)	0 (0)	0 (0)
*Socioeconomic status*					
*SEIFA IRSAD*‡*, mean (SD)*	1015.6 (81.0)	1045.1 (56.2)	1011.6 (68.9)	1003.8 (62.5)	1018.8 (74.6)
*Remoteness of home postcode*					
Major city of Australia§	87 (78.3)	20 (86.9)	14 (66.6)	11 (68.7)	14 (93.3)
Inner Regional	22 (19.8)	3 (13.0)	6 (28.5)	5 (31.2)	1 (6.6)
Outer Regional or Remote	2 (1.9)	0 (0)	1 (4.7)	0 (0)	0 (0)
Majority of respondent’s experience and perspective					
Children and young people (< 25 years)	14 (12.6)	4 (17.3)	3 (14.2)	3 (18.7)	1 (6.6)
Adults (25+ years)	35 (31.5)	4 (17.3)	7 (33.3)	7 (43.7)	5 (33.3)
Both children and young people, and adults	62 (55.8)	15 (65.2)	11 (52.3)	6 (37.5)	9 (60.0)
*Consumers’ experience of mental health*					
*Length of time experiencing mental health challenges*					
Less than 1 year	2 (2.9)	0 (0)	1 (8.3)	0 (0)	0 (0)
More than 1 year (but less than 5 years)	9 (13.2)	1 (5.5)	0 (0)	1 (9.0)	0 (0)
More than 5 years (but less than 10 years)	16 (23.5)	4 (22.2)	5 (41.6)	1 (9.0)	1 (16.6)
More than 10 years	41 (60.2)	13 (72.2)	6 (50.0)	9 (81.0)	5 (83.3)
*Accessed services for mental health, in last year*					
Yes	57 (83.8)	15 (83.3)	10 (83.3)	10 (90.9)	5 (83.3)
*Overnight stay in hospital for mental health, in last 5 years*					
Yes	24 (35.2)	6 (33.3)	6 (50.0)	4 (36.3)	1 (16.6)
*Carer/family/supporters’ experience of mental health*					
Age of person/people they are supporting (*multiple allowed*)					
0–14 years	13 (17.8)	4 (28.5)	3 (17.6)	1 (14.2)	3 (27.2)
15–17 years	13 (17.8)	2 (14.2)	4 (23.5)	1 (14.2)	3 (27.2)
18–24 years	18 (24.6)	4 (28.5)	2 (11.7)	0 (0)	5 (45.4)
25–34 years	16 (21.9)	3 (21.4)	7 (41.1)	1 (14.2)	1 (9.0)
35–44 years	11 (15.1)	1 (7.1)	2 (11.7)	0 (0)	3 (27.2)
45–54 years	8 (11.0)	2 (14.2)	2 (11.7)	1 (14.2)	0 (0)
55–64 years	5 (6.8)	2 (14.2)	0 (0)	1 (14.2)	1 (9.0)
65+ years	7 (7.6)	1 (7.1)	2 (11.7)	2 (28.5)	0 (0)
*Length of time supporting this person (/people)*					
Less than 1 year	3 (4.1)	1 (7.1)	0 (0)	0 (0)	0 (0)
More than 1 year (but less than 5 years)	23 (31.5)	5 (35.7)	5 (29.4)	3 (42.8)	3 (27.2)
More than 5 years (but less than 10 years)	13 (17.8)	2 (14.2)	4 (23.5)	0 (0)	3 (27.2)
More than 10 years	34 (46.5)	6 (42.8)	8 (47.0)	4 (57.1)	5 (45.4)
*Person they support, accessed services for mental health, in last year*					
Yes	64 (87.6)	13 (92.8)	15 (88.2)	5 (71.4)	10 (90.9)
*Person they support, overnight stay in hospital for mental health, in last 5 years*					
Yes	38 (52.0)	7 (50.0)	8 (47.0)	4 (57.1)	5 (45.4)

^*^When first culture stated was Australian, second culture stated included: British/English, Dutch, Filipino, French, German, Greek, Jewish, Malaysian, Maltese, Mauritian, New Zealander, Rural Remote culture, Tamil

^†^No participants identified as Torres Strait Islander

^‡^Socio-Economic Index for Areas (SEIFA), Index of Relative Socio-Economic Advantage and Disadvantage (IRSAD) [36]. A score is assigned for every Australian postcode on the basis of census data reflecting the economic and social conditions of people and households within an area. It has a national mean of 1000 and SD of 100, where lower scores represent greater disadvantage, and higher scores represent greater advantage

^§^Australian Statistical Geography Standard levels of remoteness [37]

### Quantitative factor analysis

Assessing against our criteria for factor extraction, initially, 28 factors had eigenvalues greater than 1.00, however, the scree plot and Horn’s Parallel analysis revealed that 4 factors (“viewpoints”) were appropriate to retain, with only 4 factors having adjusted eigenvalues greater than 1.00 (see Supplementary Material 3 for full output). The variance of the data explained by each factor (1–4) was 10.4%, 9.6%, 8.4% and 8.3%, respectively. Together, these four factors accounted for 36.7% of the variance in views for the total sample, which falls within the range of 35–40% of variance considered a sound solution [26]. Each viewpoint surpassed the criterion for having at least two significantly loading Q-sorts (factor 1, *n* = 23; factor 2, *n* = 21; factor 3, *n* = 16; factor 4, *n* = 15). Together, these four viewpoints were endorsed by 75/111 (68%) participants.

### Sub-group analyses

In sub-group analyses, two common viewpoints emerged for consumers; six for carers; and two for consumer + carers. In each age band, two common viewpoints emerged for 15–24 year olds; four for 25–64 year olds; and three for 65+ year olds. Results of the crosstabs showing how sub-group views are reflected in the main analysis are available in Supplementary Material 4. Examining the overlap of sub-group viewpoints with the viewpoints observed in the main analysis, it was determined that the common views that emerge within each sub-group were largely captured by the four common viewpoints in the main analysis. Thus, interpretation of common views was conducted on the basis of the four common views that emerge from the main analysis. One exception is that the most common viewpoint for 65+ year olds (*n* = 5/13) was not captured in the main analysis, however, the second and third most common views of people aged 65+ years old were reflected by main analysis viewpoints #1 and #4. Also of note, within the 15–24 year old age band, there were three respondents aged 15–17 years whose views were not captured in the four main viewpoints, such that the views are more reflective of people aged 18–24 years, rather than 15–24 years as intended.

### Qualitative interpretation of viewpoints

**Fig. 1:**
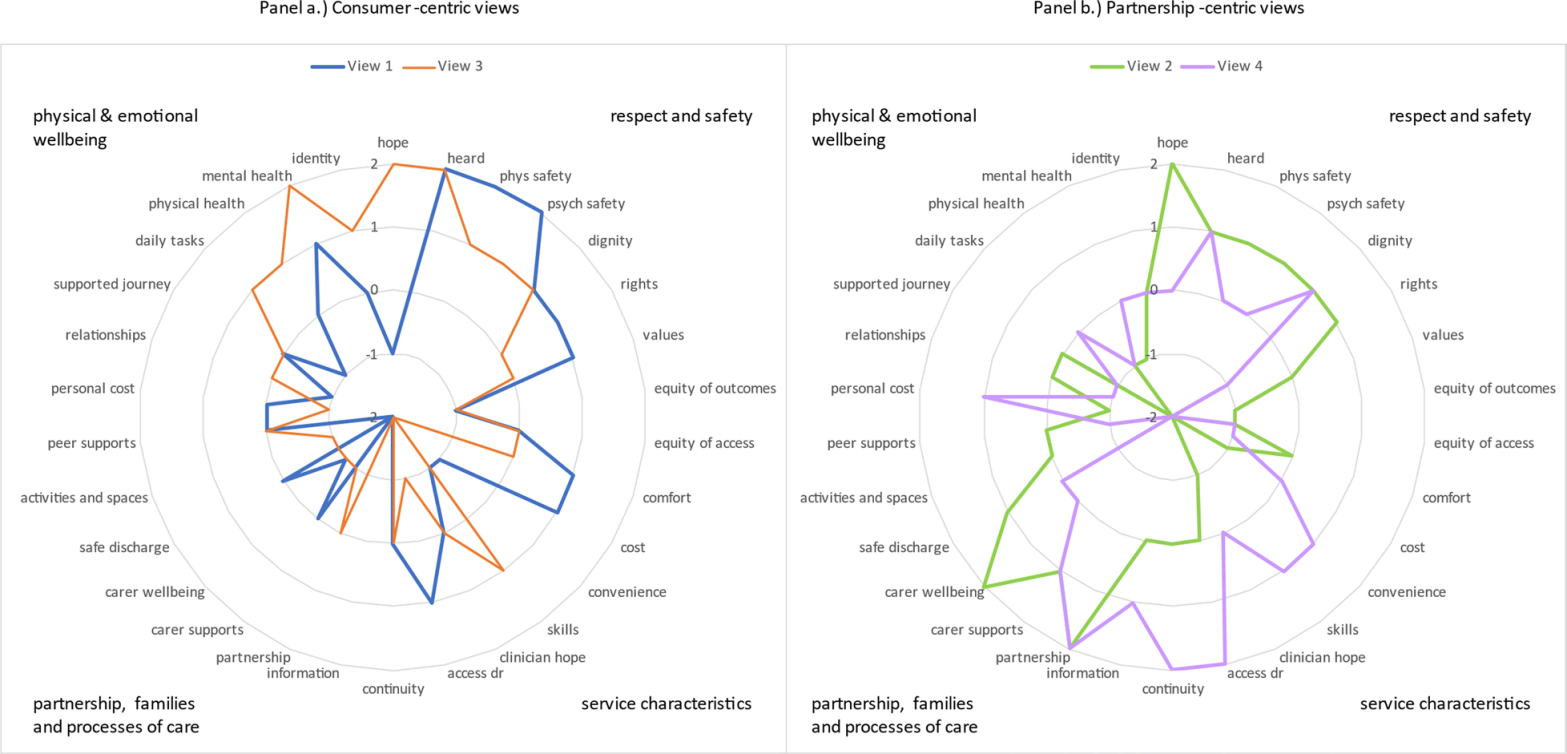
Visual representation of factor arrays for each common viewpoint. Figure 1 depicts the factor array (average representation of rankings) showing the rating for each of the 30 statements in the Q-sort for the four common viewpoints. Items rated at the outer edge (+2) were the most important, and those rated in the centre (−2) were the least important. Panel a shows the two consumer-focussed views (viewpoints #1 and #3) and panel b shows the two partnership-focussed views (viewpoints #2 and #4)

Factor arrays (representative Q-sorts), and distinguishing and consensus statements for each viewpoint are provided in Table 3. Factor arrays for each viewpoint are additionally plotted in visual format (Fig. 1) to highlight areas of difference between the viewpoints. Two consumer-centric views emerged (shown in Fig. 1a) that focus more on the emotional and physical wellbeing of the consumer, as well as elements of safety and respect. Additionally, two partnership-centric views emerged (shown in Fig. 1b) that focussed on partnership with families and carers and characteristics of the health service.

**Table 3:**
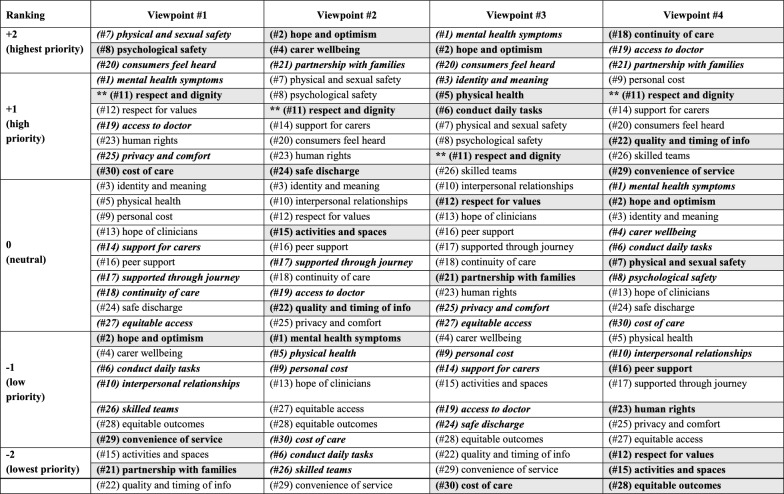
Factor arrays, distinguishing and consensus statements for each common viewpoint

The order of items within each ranking category (i.e. within each ranking category of +2, +1, 0, −1, −2) are listed in numerical order of the statement, not in order of importance. All items within the same ranking category should be considered of equal importance

*Bold italics* means distinguishing statements (rated differently to other views) according to Stephenson’s method

Bold shading means distinguishing statements according to the higher threshold of significance using Cohen’s method (note, these statements were also identified as distinguishing statements according to Stephenson’s method)

^**^ Bold shading means one consensus statement (rated equally across views) according to Cohen’s method

#### Consensus statements

One consensus statement was identified, where all four views rated *(#11) respect and dignity* as +1 important to measure (Table 3). Also rated highly by all four views was *(#20) consumers feel heard.* These two statements were the only two rated as +1 important or +2 most important by all four views. Consumers and carers spoke about these concepts in their own words, referencing how integral they are to feelings of hope and continued recovery:

“*Consumers must be treated as experts of their own lives and respected as partners in their treatment and recovery. Without being listened to, and being heard, there is no foundation for respect or a relationship with their treating team and will lead to disengagement and loss of hope*” [ID76, consumer + carer].

“*People are treated with respect and their views are listened to, including their concerns. This requires recognizing and addressing the power imbalance between medical professionals and consumers/carers. They feel empowered to ask questions, and express doubts about how and when they are treated. This also means a non-judgmental environment that fosters kindness and care. For example, consumers shouldn't feel that they are objects or weirdos with mental illness, rather, they should be seen as any other person*” [ID95, carer].

“*When I am being seen as an illness, majority of the time, all respect for me as a person is overlooked, plus being seen as an illness means that my dignity is not valid or taken into consideration, this includes my rights are ignored, I am not heard or felt listened to, which then leads to more distress for me and then it becomes a vicious cycle. But being seen as a person, gives me the feelings of I am a person who has mental health problems rather than I am a problem*” [ID62, consumer].

At the other end of the scale, several statements were consistently rated as neutral or lower importance by all four views: *(#10) interpersonal relationships; (#13) hope and optimism of clinicians; (#15) healing activities and spaces; (#16) peer support; (#17) supported through journey; (#27) equitable access*; and *(#28) equitable outcomes*. The interpretation of these neutral/low ratings, however, requires caution. Some participants reported that they selected the least important things on the basis of being “*too difficult and too ambiguous to measure*” [ID81, consumer + carer]; “*an assumption that services are already asking and measuring [these items]*” [ID87, carer]; that they “*discounted one if I felt it had been covered by another statement*”[ID4, carer]; or were considered important but “*beyond the focus of acute care”*[ID40, carer]. Many said they found it difficult to choose the least important items, as they were all important. It is therefore recommended to not regard these items as low importance, but rather that other items were placed ahead of these within the constraints of the response format. Nevertheless, the process of asking participants to identify lower important items has positively contributed to the overall quantitative ratings and the ability to identify most important items.

#### Viewpoint #1: safety before all else

This view was characterized by placing the highest priority on statements related to *(#7) physical and sexual safety (*+*2), (#8) psychological safety (*+*2) and (#20) consumers feel heard (*+*2)*. Compared with other views, viewpoint #1 placed greater emphasis on elements related to safety as the essential “*first step to feel safe before any treatment would work*” [ID19, consumer + carer]. Physical, sexual and psychological safety were also described as a priority to prevent further harm: “*without physical and sexual safety in the healthcare setting, the consumer is at risk of being further harmed thereby exacerbating any existing mental health issue, compounding trauma, increasing distrust of healthcare, decreasing help seeking ability of the consumer*” [ID76, consumer + carer]; “*neglecting consumers psychological safety can derail the healing process and even cause further harm*” [ID24, consumer].

People who held viewpoint #1 also prioritized *(#30) cost of care (*+*1)* more than other views, stating for example: “*cost is a huge thing for many people. It is a barrier to get help and the support they need from professionals. Money can be taken for granted when you have it, but when you experience not having it, it puts you into the perspective of how so many people struggle with this barrier*” [ID72, consumer]; “*money should never be the deciding factor in someone recovering*” [ID21, consumer + carer].

Compared with other views, people who held this view placed lower priority on *(#21) partnership with families (−2)*. Carers who held this view focussed on the consumer’s recovery as the priority: “*as a carer, my priority is whom I’m caring for so hopefully they will be able to cope without such a great need of a carer or support*” [ID 66, carer]; “[family partnership] *would be nice, but I don’t care as long as they treat the consumer*” [ID88, carer].

This viewpoint was the most likely to be shared across all sub-groups: held as a common viewpoint by consumers (*n* = 9/23), carers (*n* = 5/23) and consumer + carers (*n* = 9/23). In sub-group analyses, this view was the most commonly held for consumers, consumer + carers and adults aged 25–64 years; and was the second most common viewpoint for carers, young people aged 15–24 years, and people aged 65 + years.

#### Viewpoint #2: hope and partnership in processes of care

This view was characterized by placing the highest priority on statements related to *(#2) hope and optimism of consumer/carer (*+*2), (#4) carer wellbeing (*+*2) and (#21) partnership with families (*+ *2)*. Compared with other views, people who held viewpoint #2 placed a greater priority on partnership with families and the wellbeing of carers, recognizing the network of people and interactions surrounding consumers rather than a single episode of care. This viewpoint can be captured through the words of one carer, who said:

“*People don't live in a vacuum. We need to move away from an individualistic approach to one that considers the person as part of a network of family, friends and other connections. Therefore, mental health services need to involve, support and work with families and carers and view them as partners in care. It is also important to recognize how families and carers are impacted, and that health services need to support them so that these carers can provide the best possible support to the consumer while also consider[ing] their own needs” [ID69, carer].*

People who held this viewpoint were also more likely to place higher importance on processes around *(#24) safe discharge (*+*1)*, referencing this as a key period for partnership with families and carers: “*there has to be a realistic and supported plan for discharge. Otherwise people just end up back in the service*” [ID2, consumer + carer]; “… *as recovery advances the next level might be … about helping consumer[s] help themselves back outside the clinical setting – at the moment it’s like a steep cliff once [consumers] leave clinical setting/hospital so discharge [is] not so effective (a failed discharge just makes things worse*)” [ID45, consumer + carer].

Of all the views, people who held this view placed a lower priority on characteristics of the service such as *(#30) cost of care (−1)* and *(#29) convenience of service (−2)*, stating “*I’d pay anything for my child’s life, yes cost, it’s a barrier, but not as important as the others*” [ID98, carer]; “*distance, cost, doesn’t matter. Health does*” [ID33, consumer + carer]; and “*the location of the service isn’t that important; consumers can make that work as long as they can access good quality care*” [ID31, consumer].

This viewpoint was predominantly held by carers (*n* = 9/21), and consumer + carers (*n* = 8/21) though was shared by some consumers (*n* = 4/21). This view represented the most commonly held viewpoint for carers in sub-group analyses, and was also amongst the most common views for consumers and consumer + carers. This view was held by adults (*n* = 16/21) and younger people (*n* = 5/21), but none aged 65+ years.

#### Viewpoint #3: physical and emotional health and wellbeing

This view was characterized by placing the highest priority on statements related to *(#1) mental health symptoms (*+*2), (#2) hope and optimism of consumer/carer (*+*2) and (#20) consumers feel heard (*+*2).* Compared with other views, viewpoint #3 placed greater priority on the physical and emotional wellbeing of consumers, including *(#2) hope and optimism of consumer/carer (*+*2)*, *(#3) identity and meaning (*+*1), (#5) physical health (*+*1)* and *(#6) conduct daily tasks (*+*1)*. “*These statements are about the consumers experience of their recovery journey*” [ID48, consumer]; “*everyone needs to get out of bed in the morning feeling that they have purpose and meaning in their lives and hope that they can be well and be active in their community without judgement*” [ID22, consumer]; “*it is possible to live well with mental illness if you are supported to maximize your functional ability and maintain important relationships. A mental health service team must be able to deliver evidence informed clinical treatment but also the complimentary interventions that support and sustain recovery*” [ID99, consumer + carer].

Compared with other views, people who held this view placed lower priority on *(#21) partnership with families (0)*: “*the consumer is to be put at the top of the list*” [ID54, consumer]; “*I don’t feel like families and carers should be prioritized. I understand that they need to be supported and also that they can help their family member who has mental illness, but I don’t think they are the top priority*” [ID35, consumer].

This viewpoint was predominantly held by consumers (*n* = 9/16), though was shared by some carers (*n* = 5/16) and consumer + carers (*n* = 2/16). This view was held predominantly by adults (*n* = 15/16), with only one younger person (*n* = 1/16) and no one aged 65+ years holding this view.

#### Viewpoint #4: care access, continuity and partnership with families

This view was characterized by placing the highest priority on statements related to *(#18) continuity of care (*+*2), (#19) access to doctor (*+*2) and (#21) partnership with families (*+*2)*. Viewpoint #4 also placed a high priority on processes of care and service characteristics that other views regarded as neutral or lower importance, such as *(#22) quality and timing of info (*+*1); (#26) skilled teams (*+*1); (#29) convenience of service (*+*1)*; and *(#9) personal cost (*+*1)*.

People who held this view spoke frequently about the people surrounding the consumer: “*working with carer[s and] families is a vital piece of the puzzle… It takes a tribe and village to support those with mental health and [have a] holistic approach*” [ID71, consumer + carer]; “*differently trained professionals have different help to offer…To meet the complex needs of a consumer, we need an appropriate mix of professionals.*” [ID8, carer]. Continuity of care was also important: “*need to look around for suitable clinicians… Working together towards the outcome.*”[ID7, carer]; “*care from start, during and end. Consistent approach [and] goal between treating team. Same vision and strategies, same rules and messages*” [ID71, consumer + carer].

People who held this view gave neutral ratings to elements that were strongly important in other views, for example, *(#30) cost of care (0); (#7) physical and sexual safety (0); (#8) psychological safety (0); (#2) hope and optimism of consumer/carer (0); (#4) carer wellbeing (0)* and *(#1) mental health symptoms (0).*

This viewpoint was predominantly held by carers (*n* = 9/15). While some consumers (*n* = 4/15) and some consumer + carers (*n* = 2/15) held this view, this did not emerge as a commonly held viewpoint of consumers in sub-group analyses. This view was held predominantly by adults (*n* = 12/15) and older people (*n* = 2/15), with only one younger person holding this view (*n* = 1/15).

## Discussion

Four common views emerged from the quantitative factor analysis: (1) Safety before all else, prioritizing physical, sexual and psychological safety; (2) hope and partnership in processes of care; (3) physical and emotional health and wellbeing; and (4) care access, continuity and partnership with families. Key priority areas that were common to all views were the high priority placed on having an environment in the health service that fosters respect and dignity and that consumers feel heard and listened to. Each of the four views was characterized by placing a different level of priority on additional areas such as: physical, sexual and psychological safety and costs of care (viewpoint #1); hope; partnership with families; carer wellbeing; and safe discharge processes (viewpoint #2); mental health symptoms; physical health; daily tasks (viewpoint #3); continuity of care; access to doctors; information provided to families; and personal costs of care (viewpoint #4). In sub-group and qualitative analyses, differences were observed regarding the likelihood of consumers and carers holding each of the views; where viewpoints #1, #2 and #3 were largely shared by both consumers and carers and viewpoint #4 was predominantly held by carers. Differences were also observed by age group; the views of people aged 15–17 years were not captured in the four most common views, and people age 65+ years were more likely to hold viewpoints #1 and #4 but not viewpoints #2 or #3.

Key priority areas identified in our study were also identified in the previous qualitative study conducted in the United Kingdom [19], including person-centred care, person-specific aims and outcomes, healthcare environment, communication and minimizing risk. These concepts were prioritized by both consumers and carers in our study, each to varying degrees. Items that were considered important in the United Kingdom study, such as access to care, coordinated care, continuity of care and capacity and resources of the care team, were only highlighted by one view, viewpoint #4, predominantly held by carers in our study. Our findings build upon the United Kingdom study [19], by describing these concepts in greater detail, and identifying the priorities of consumers and carers separately. The alignment of our findings with the United Kingdom study suggests that our results may be relevant for broader mental healthcare settings.

Our qualitative results suggest that younger people aged < 18 years and older people aged 65+ years may view concepts of value and outcome measurement in mental health differently to adults aged 25–64 years. We noted there were three respondents aged 15–17 years whose views were not captured in the four main viewpoints. Similarly, only 4 of 13 people aged 65+ years held one of the common views, with two common views not held by any participant aged 65+ years. This suggests a need to examine these more targeted age sub-groups in further detail in future research to better capture these views, as this has not been previously explored.

Participants in our study frequently communicated that it was difficult to choose between the presented options for measurement. Participants in previous priority-setting studies (for example eliciting research agenda priorities [20, 21]) have similarly commented they found it challenging to prioritize some topics over others, finding them all equally important. Perhaps, in line with these previous studies, the difficulty of prioritizing measures in our study might indicate that including any of the more highly rated outcomes in future value-assessment frameworks would address a significant gap in the way we define and measure value in mental healthcare.

Strengths of the study are that we used an established methodology to identify and describe the priorities of consumers and carers in mental healthcare. To our knowledge, this study provides the first insight into the similarities and differences in the views of consumers and carers regarding their priorities for value assessment and measurement in mental healthcare, and the first insight into differences in these views by age group. Limitations include that our sample was largely English-speaking, non-indigenous Australians, with higher education attainment, and living in a major city. Thus, the findings of our current study may not reflect the views of people from different socio-economic and cultural sub-groups. Additionally, due to the way participants were recruited, the views of consumers and carers included in the analysis may not be representative of people who are not already engaged with peak bodies, who may be less well-versed in the topics being discussed.

### Implications, recommendations and future research

This work contributes new evidence within international literature regarding how to define and measure value in healthcare [4, 5, 35]. While traditional value frameworks based on costs and QALYs provide an important starting point, mental healthcare presents a uniquely complex and widespread burden, and therefore holds potential to create meaningful value that is not fully captured by current frameworks. Emerging value assessment frameworks, such as the The Professional Society for Health Economics and Outcomes Research (ISPOR) value flower [8], suggest value assessment in healthcare should include consideration of costs and QALYs as well as wider areas of impact such as productivity; family spillovers; value of knowing; insurance value; fear of contagion; severity of disease; value of hope; real option value; equity; and scientific spillovers. Our results reveal that some of these aspects are valued by consumers and carers in mental healthcare (in particular, hope and family spillovers). However, there are aspects missing from this conceptual framework that apply uniquely to mental healthcare. Most importantly, the concepts of physical and psychological safety; fostering an environment of respect and dignity; and that consumers feel heard and listened to. These concepts were strongly prioritized by consumers and carers in our work, and were also common themes emerging from the Royal Commission into Victoria’s Mental Healthcare System [3]. No existing international value assessment framework has yet been developed specifically for use in mental healthcare [18], however, there is scope for this to become a priority for development. Our results make steps towards identifying several mental health-specific attributes to include in such a framework.

In real-world decision-making, various mental healthcare options under consideration may rate differently on the measures that consumers and carers value. This means that decision-making, in practice, may involve making trade-offs between these outcomes and aspects of mental healthcare. To aid policy decision-making based on our results, future research should examine the relative weightings – or the relative value – of each measure in a manner that would guide priority setting in this area. Such weights would reflect the relative value of each outcome on the basis of the preferences of consumers and carers.

### Conclusions

The results of this study provide an insight into the key priority areas for consumers and carers regarding the most important measures to include in health economic evaluations which asses the incremental value of competing options in mental health care. Shared priority areas for both consumers and carers included: having an environment of respect and dignity; that consumers feel heard and listened to; physical, sexual and psychological safety; a sense of hope; partnership with families; and measures of physical and emotional health and wellbeing. Accurately capturing and including these outcomes and aspects of mental healthcare in evaluation frameworks would provide a way of focussing mental healthcare decision-making on the attributes that are of most value to people with lived experience, thereby addressing important shortcomings in current decision-making processes.

## Supplementary Information

Supplementary material 1.

## Data Availability

An anonymized dataset generated and analysed during the current study is available from the corresponding author on reasonable request.
