# Supplementary material for: What are the priorities of consumers and carers regarding measurement for evaluation in mental healthcare? Results from a Q-methodology study
Source: Health Res Policy Syst. 2024 Nov 11;22:150. doi: 10.1186/s12961-024-01239-y (PMC11552116; doi:10.1186/s12961-024-01239-y)
Supplement: Supplementary file 1 — Supplementary material 1. [file 12961_2024_1239_MOESM1_ESM.docx]

**SUPPLEMENTARY MATERIAL**

**What are the priorities of consumers and carers regarding measurement for evaluation in mental healthcare? Results from a Q-methodology study.**

**Running Title**: Consumer and carer priorities in mental healthcare.

**Table of Contents**

[**Supplementary Material 1.** Complete online survey. 2](#_Toc153196473)

[**Supplementary Material 2.** The Q-sort distribution grid. 23](#_Toc153196474)

[**Supplementary Material 3.** Output from Principal component analysis: Determining the number of factors to be extracted. 24](#_Toc153196475)

[**Supplementary Material 4.** Results of subgroup analysis and crosstabs for consumers and carers and each age group separately. 30](#_Toc153196476)

# **Supplementary Material 1.** Complete online survey.

Complete Online Survey

**** Note to readers:**

****red text** represents notes and are not seen by participants. All other text is visible within the online survey.

**** SURVEY COVER PAGE STARTS HERE ****

| What does good value in mental healthcare mean to you?  When changes are made to the mental healthcare system, we need to evaluate these changes by measuring some important outcomes.  This helps us decide if the change has made an improvement, and is good value.  There a lots of things that would be great to measure,  but we need to start to prioritise these - to capture the most important things.  In this research study, we want to know what YOU think should be measured when changes are made to the mental healthcare system. |
| --- |

☐ Enter Survey ***[**proceeds to Plain Language Statement, below]***

Section 1: About this study

Please take a few minutes to watch this video that will go through some important information about this research study before you choose whether to participate.

[Embedded video: PICF]

How can I take part in the survey?

You may like to discuss this research project with a trusted family member or support person before you choose to participate, and you can have this support person with you while you complete the survey.

If you **do not wish to take part**, simply close this window.

If you **choose to take part**, please download and read through the Plain Language Statement pdf. [link to online PDF to download]. You should keep a copy of this for your records.

Consent Statement:

- I consent to participate in this project, the details of which have been explained to me, and I have been provided with a written Plain Language Statement to keep.
- I confirm I am aged 15 years or older.
- I understand that the aim of this research is to investigate the outcomes that matter the most to consumers and carers, when we measure the value of changes to the mental healthcare system.
- In this project I will be required to complete a one-time online survey.
- I understand that my participation in this project is for research purposes only.
- I acknowledge that the possible effects of participating in this research project have been explained to me.
- I understand that my participation is voluntary – I can choose whether to participate or not. I am free to withdraw from this project anytime without giving a reason, and that my choice will not have any effect on my existing clinical care (if any).
- I understand that the data from this research will be stored at the University of Melbourne and will be destroyed 15 years after publication, in line with standard research data processes.
- I understand that this survey is anonymous (the research team will not know my name), and the information I provide will remain confidential subject to any legal requirements; my data will be password protected and accessible only by the named researchers.
- I understand that after I complete this consent form, it will be retained by the researchers.

Click the box below to provide your consent, and continue to the survey.

☐ I consent to participate in the study.

 ☐ Next ***[**proceeds to ‘Before we start’, below]***

Before we start

**A note about the language used in this survey**

We recognise that language is deeply powerful. We have worked with lived experience advisors to develop this survey, so that we are using language that is person-centred and recovery-focused. However, we know that you may not personally identify with the terms that are used.

***Consumers***
There are a number of terms used to refer to people accessing mental health services. This can include consumers, clients, service users and patients. In this survey, wherever possible, we use the terms**‘person’, ‘people with lived experience’** and **‘people accessing mental health services’**. We use the term **‘consumer’** when we need to make a distinction between different people or roles – for example consumer and carer (knowing that someone can be both of these roles). This can also refer to people in recovery, or who have previously accessed services for a mental health problem.

***Carers/families/supporters***
We know that many people never think of themselves as carers – they feel they are doing what anyone else would in the same situation – looking after their family member, partner or friend. We also recognize that many people do not identify with the term ‘carer’ and the kind of relationship this term denotes. In this survey, we use the terms **‘carer/family member/supporter’** to recognize the many ways that this relationship can exist. We use these terms to refer to anyone – biological family, family of choice, partner(s), support person, or friend(s) – of someone with a mental illness, whose life is also affected by that illness.

Mental health and wellbeing support services

If you are feeling distressed or upset right now, or at any time during the survey, we encourage you to close the survey, and to contact one of the below support lines.

- Lifeline (24 hours): phone 13 11 14 or [www.lifeline.org.au](http://www.lifeline.org.au/)
- Beyondblue: phone 1300 224 636 or [www.beyondblue.org.au](http://www.beyondblue.org.au/)
- SANE Australia: phone 1800 187 263 or [www.sane.org](http://www.sane.org/)
- Kids Helpline (24 hours): phone 1800 55 1800 or [www.kidshelpline.com.au](http://www.kidshelpline.com.au)
- Tandem Carers support and referral line: phone 1800 314 325 or [tandemcarers.org.au](http://www.tandemcarers.org.au/)

If you are currently experiencing a mental health crisis, or if feel you are at risk of harming yourself or others, participating in this research study may not be in your best interests, and you should immediately seek support.

☐ Next ***[**proceeds to ‘A little about you’, below]***

Section 2: A little about you

Please answer these questions that will tell us a little about you.

These questions are important for us to know about the range of people that have completed this survey.

**Below are the groups of people that we would like to hear from in this research.**

2.1 **Which group(s) listed below do you identify with?** *(Tick all that apply)*

☐ I’m a person **with lived experience of mental health challenges** (a ‘**consumer**’ as defined on the previous page)

☐ I’m a **carer, family member or supporter** of someone with mental health challenges (as defined on the previous page)

☐ None of the above ***[End Survey]***

2.2. **How old are you?**

☐ 0-14 ***[End Survey]***

☐ 15-17

☐ 18-24

☐ 25-34

☐ 35-44

☐ 45-54

☐ 55-64

☐ 65 and over

2.3 What gender do you identify with? [*Please feel free to self-describe any option you choose below*]

☐ Female

☐ Male

☐ Non-binary

☐ Other, *please provide in your own words:* | [*free text box*] |

☐ Prefer not to say

2.4 **What is your home postcode?**

☐☐☐☐

2.5 **What is your cultural and ethnic background?** (For example: Chinese; English; Australian; Maori; Scottish; Sikh; Syrian etc). Consider your own, your parents and grandparents background. Provide at least one in your own words.

☐ 1^st^ | [*free text box*] |

☐ 2^nd^ (if any other) | [*free text box*] |

☐ 3^rd^ (if any other) | [*free text box*] |

2.6 **What is the main language you speak at home?**

☐ English

☐ Other, *please specify in your own words:* | [*free text box*] |

☐ Prefer not to say

2.7 **Are you of Aboriginal or Torres Strait Islander descent?**

☐ Yes, Aboriginal

☐ Yes, Torres Strait Islander

☐ Yes, both Aboriginal and Torres Strait Islander

☐ No

☐ Prefer not to say

2.8 **What is the highest level of education you have completed?**

☐ Bachelor Degree or above

☐ Certificate I / II / III / IV (including trade certificate)

☐ Completed Year 12 (or equivalent)

☐ Some school, but not completed Year 12 (or equivalent)

☐ Never attended school

☐ Still at school (in high school)

☐ Prefer not to say

***** The following questions appear according to the box(es) ticked in 2.1.***

***** These additional questions appear if participant group ticked above = consumer***

#### Now, thinking about your own mental health:

2.9.1 **For how long would you say you have you been experiencing mental health challenges?**

☐ Less than 1 year

☐ More than 1 year (but less than 5 years)

☐ More than 5 years (but less than 10 years)

☐ More than 10 years

2.9.2 **Have you accessed any services for mental health support in the last year?** (This could include your GP, a psychologist, or psychiatrist, paediatrician, an emergency department or hospital for mental health reasons, or Headspace, Orygen, or another mental health service.)

☐ Yes

☐ No

2.9.3 **Have you had an overnight stay in hospital for mental health reasons in the last 5 years?**

☐ Yes

☐ No

***** these questions appear if participant group ticked above = carer***

#### Now, thinking about your role as family/carer/supporter:

2.9.4 **How old is the person you care for/support (not yourself)?**

☐ 0-14 years old

☐ 15-17

☐ 18-24

☐ 25-34

☐ 35-44

☐ 45-54

☐ 55-64

☐ 65 years and over

☐ I care for or support multiple people with mental health difficulties – please list all the ages in the box:

| [*free text box*] |

2.9.5 **For how long have you been caring for/supporting someone with mental health difficulties?**

☐ Less than 1 year

☐ More than 1 year (but less than 5 years)

☐ More than 5 years (but less than 10 years)

☐ More than 10 years

2.9.6 **Has the person you care for/support accessed mental health services for support in the last year?** (This could include their GP, or a psychologist, or psychiatrist, paediatrician, or attended an emergency department or hospital for mental health reasons, or Headspace, Orygen, or other mental health service.)

☐ Yes

☐ No

2.9.7 **Has the person you care for/support had an overnight stay in hospital for mental health reasons in the last 5 years?**

☐ Yes

☐ No

#### We know that people come from many different experiences in child and adult mental healthcare. *Please select which applies best to you.*

2.10**. From my experience and in my views, I’m thinking more about mental healthcare for:**

☐ Children and young people (aged less then 25 years)

☐ Adults (aged 25 or older)

☐ Both – children and young people, and adults

- Lifeline (24 hours): phone 13 11 14 or [www.lifeline.org.au](http://www.lifeline.org.au/)
- Beyondblue: phone 1300 224 636 or [www.beyondblue.org.au](http://www.beyondblue.org.au/)
- SANE Australia: phone 1800 187 263 or [www.sane.org](http://www.sane.org/)
- Kids Helpline (24 hours): phone 1800 55 1800 or [www.kidshelpline.com.au](http://www.kidshelpline.com.au)
- Tandem Carers support and referral line: phone 1800 314 325 or [tandemcarers.org.au](http://www.tandemcarers.org.au/)

 ☐ Next ***[**proceeds to Practice Task, below]***

Section 3: Practice task

Please take a couple of minutes to watch this video that explains how to complete the ranking task on the next page.

**It is important that you watch this before continuing.**

[Embedded video Survey Instructions v3: How to complete the ranking task]

3.2. **I have watched this instruction video, and I understand the task.**

☐ Yes

☐ No

(***If no***: ***this message appears:*** Please watch the video before continuing. If you have any questions about how to complete the survey, you can contact the research team via email [*email link*])

 ☐ Next ***[**proceeds to Main Ranking Task, below]***

Section 4: What should we measure to understand ‘good value’ in mental healthcare?

**This is the most important part of this survey.** **Please think carefully about your answers, and take as much time as you need. You can save your answers and come back if you need to.**

***“What do YOU think are the important outcomes we should measure to understand if a change to mental healthcare is ‘good value’?”***

| ***“We should measure if the change has impacted...”***  ** Bold parts of the statements listed here were shown on screen to the participants. Lighter text following the “(?)” was available as extra information for each item when hovering over the “(?)” on screen.* | **LOW PRIORITY**  **Mark the 10 least important items to measure)** | **HIGH PRIORITY**  **Mark the 10 most important items to measure** |
| --- | --- | --- |
| 1. **Consumers’ experience of mental health symptoms.** (?) This can include changes in the severity of the symptoms. | ☐ | ☐ |
| 1. **Consumers’, and family/carer/supporters’ sense of hope and optimism.** (?) This can include hope that personal recovery is possible, contemplating change, finding support for motivation to make change happen, positive thinking, and having dreams and aspirations for the future. | ☐ | ☐ |
| 1. **Consumers’ sense of identity and meaning**. (?) This can include a positive sense of self, overcoming stigma, having self-confidence, a sense of meaning and contribution, realising your abilities. | ☐ | ☐ |
| 1. **The family/carer/supporter’s overall wellbeing.** (?) This can relate to all aspects of life, such as having a good diet, a sense of belonging, a sense of meaning and contribution, realising your abilities, having safe housing, having goals and dreams, feeling able to cope with stresses of life, enough money, someone to talk to for support in their caring role. | ☐ | ☐ |
| 1. **Consumers’ physical health**. (?) Including short or long-term health problems, their level of pain, and having a healthy diet. | ☐ | ☐ |
| 1. **Consumers’ ability to conduct daily tasks.** This includes how well they can move around and care for themselves, their ability to conduct tasks such as self-care, food preparation, house-keeping, feeling able to cope with the stresses of life. | ☐ | ☐ |
| 1. **The sense of physical and sexual safety in the healthcare setting.** (?) This includes safety from actions they themselves or others may make, including acts of physical or sexual violence. This could be real or perceived safety. | ☐ | ☐ |
| 1. **The sense of psychological safety in the healthcare setting and in interactions with staff.** (?) This includes whether consumers and families/carers/supporters feel empowered to voice their suggestions or concerns, and to build on their strengths, make informed choices and play a central role in their health and other aspects of life. To do this without fear of interpersonal, professional or social consequences. | ☐ | ☐ |
| 1. **The personal and economic impacts of accessing care (eg. Safe housing, relationship, jobs), for consumers, families/carers/supporters**. (?) This includes impacts such as losing a job, impacts on relationships, housing or family, or – on the other hand – having access to safe housing, enough money, access to employment and education opportunities. | ☐ | ☐ |
| 1. **Consumers’ relationships with those who are important to them as well as others.** (?) This can include their sense of belonging, and the quality of relationships with their family, friends, partners, or with school/work colleagues. Recognising that it is often not possible to separate a person’s personal recovery journey from their roles and relationships within the family. Placing the family at the centre of recovery. | ☐ | ☐ |
| 1. **An environment in the health service that fosters respect and dignity.** (?) This includes shared ownership of the environment that people are in – consumers, families/ carers/ supporters and staff. This could include many things including the way the staff, consumers and families/carers/supporters treat one another; the attitudes of the staff; the language that is used in a service; and the physical environment of the healthcare setting. | ☐ | ☐ |
| 1. **The level of respect and understanding shown for personal values that are important to consumers and families/carers/supporters, including connection to culture, faith based and/or spiritual values; and gender identity**. (?) This can be related to the level of consideration given to cultural values and beliefs, or in physical or spiritual connection to land and community; or related to faith-based or spirituality traditions and protocols; or the use of pronouns and respect for gender identity. | ☐ | ☐ |
| 1. **A culture of hope and optimism in the care provided by the clinician(s) and professional staff**. (?) This includes the sense of hope and positivity of the clinician, and the attitudes and expectations they have for the outcomes of the care provided. | ☐ | ☐ |
| 1. **The way that families/carers/supporters are supported by the health service**. (?) This includes whether opportunities for family/carer partnership are explored; and referrals to carer specific support are offered and facilitated (if desired). This can include linking in with the Carer Consultants and Carer Peer Workers within the service. Whether families/carers feel they are supported to have the right mix of skills and capabilities to meet consumers’ needs. | ☐ | ☐ |
| 1. **Access to healing activities, spaces and places**. (?) Including whether there were activities that were available that suit consumers’ and families/carers/supporters’ needs and preferences, and access to outdoor spaces. | ☐ | ☐ |
| 1. **Access to peer support throughout the journey with mental health services**. (?) Including access to information about peer workers, referral to consumer programs, advocates. | ☐ | ☐ |
| 1. **How supported consumers feel to continue their recovery journey.** (?) This can include whether consumers are included as partners and are empowered in planning their care; having access to care options; and whether consumers feel supported to follow the steps that they have planned. | ☐ | ☐ |
| 1. **The continuity of care experienced.** (?) This can include having the same clinicians, or the way that the care between different teams or services is coordinated; not having to repeat yourself to different staff. | ☐ | ☐ |
| 1. **The level of access to the treating doctor or psychiatrist when needed**.  (?) This can include whether staff made an effort to see consumers or carers/families/supporters when they wanted to be seen. | ☐ | ☐ |
| 1. **Whether consumers feel listened to, and feel heard**. (?) This could include partnering with consumers in planning care and treatment options; having consumers’ preferences and wishes driving their care; being listened to in all aspects of care. | ☐ | ☐ |
| 1. **A sense of partnership and listening to families/carers/supporters throughout the journey with mental health services**. (?) This means working with families/carers/supporters, listening to their preferences, and having a two-way flow of information. Whether carers/families/supporters feel listened to, and feel heard and believed. Acknowledging the knowledge and expertise of carers/families/supporters. | ☐ | ☐ |
| 1. **The quality and timing of information provided to families/carers/supporters about their role, and the ongoing recovery of the consumer.** (?) Taking into account policies related to privacy and disclosure of health information, that carers/families/supporters are provided with the right amount of information on their role, and the ongoing care, treatment and recovery for the consumer. This includes having information in plain language at multiple times during the journey. | ☐ | ☐ |
| 1. **Whether consumers' and families/carers/supporters' human rights are upheld.** (?) This includes emphasis on advance statements, supported decision making, second psychiatric opinion, avenues for complaints, nominated persons, the right to communicate, legal and non-legal advocacy, visitor and supporter access, and staff recognising people’s human rights in their staff huddles/meetings. | ☐ | ☐ |
| 1. **Families and consumers experience discharge that is informed, supported and sustainable.** (?) This includes having access to safe accommodation, services that are reaching out, and consumers’ and families’ level of knowledge of services and strategies if required in the future. | ☐ | ☐ |
| 1. **Consumers’ privacy and physical comfort in the service.** (?) Here, this means their physical privacy, their own space, and the physical environment of the service/healthcare setting, and the accommodation furnishings. | ☐ | ☐ |
| 1. **Whether (paid) care teams have an appropriate mix of skills and capabilities for consumers’ needs.** (?) This can include the types of skillsets and disciplines of staff that partner with consumers: peer workers, consumer/carer consultants; access to wholistic care; inclusion of supports and treatment related to alcohol and other drugs, if appropriate. | ☐ | ☐ |
| 1. **Whether the service provides better access for those who need it most.** (?) This means the equity of the access to care; better access for people who need it most, or better serving the most marginalised people. | ☐ | ☐ |
| 1. **Whether the service provides more equal (or equitable) outcomes for people accessing care.** (?) This means improving outcomes for certain groups of people who would otherwise have worse outcomes than others; the equity of the outcomes of the care provided. | ☐ | ☐ |
| 1. **The convenience and accessibility of the location** **of the service**. (?)Such as being close to family and friends, or public transport, parking or other community services. Including whether the service is available in the regions where it is most required (rather than all being in the middle of the city, for example). | ☐ | ☐ |
| 1. **The cost – the amount of money consumers, and families/ carers/ supporters have to pay for care.** (?) This means money they have to pay, or money they save due to accessing care. | ☐ | ☐ |

☐ Next ***[**proceeds to Stage 2 of ranking task, below]***

Section 4.2: Ranking (cont.)

The following questions are based on your rankings in the previous page. If these do not feel right for you, you can go back and edit your responses before continuing.

**Firstly, thinking about the HIGHEST priority items you selected**On the previous page, you selected these statements as the 10 MOST important to measure. Now, please select the 3 items that you think are the most important in this list. (The ones you would rank at the TOP or the HIGHEST overall.)

| **The very HIGHEST priority items are:** | **HIGHEST PRIORITY**  (mark the 3 MOST important items to measure) |
| --- | --- |
| ***{ *** the options listed here are the 10 x statements that participants ranked as the HIGHEST priority in the ranking task on the previous page *** }***  ***1.***  ***2.***  ***3.***  ***[…]***  ***9.***  ***10.*** | ☐  ☐  ☐  ☐  ☐  ☐ |

**Now, thinking about the LOWEST priority items you selected**On the previous page, you selected these statements as the 10 LEAST important to measure. Now, please select the 3 items that you think are the least important in this list. (The ones you would rank at the BOTTOM or the LOWEST overall.)

| **The very LOWEST priority items are:** | **LOWEST PRIORITY**  (mark the 3 LEAST important items to measure) |
| --- | --- |
| ***{ *** the options listed here are the 10 x statements that participants ranked as the LOWEST priority in the ranking task on the previous page *** }***  ***1.***  ***2.***  ***3.***  ***[…]***  ***9.***  ***10.*** | ☐  ☐  ☐  ☐  ☐  ☐ |

☐ Next ***[**proceeds to ‘In Your Own Words’, below]***

**Well done! You're on the last page of questions!**

Section 5: In your own words

**The following questions will help us to understand more about your personal views.**

**Please do not include any specific details of your own experience of care in your responses on this page**. We ask this because we are unable to provide individual support for you, and these discussions should be held in a supportive space for you. If you feel that you would like to discuss your personal experiences, we encourage you to talk to a trusted family member or friend, or consider contacting one of the support services listed on this page.

- 1. You rated these statements as ‘the MOST important to measure’:

{*feed in 3 statements from Stage 2 of ranking task*}.

What do these statements mean to you, and why do you feel strongly these should be measured?

[*free text box*]

- 1. You rated these statements as ‘the LEAST important to measure’:

{*feed in 3 statements from Stage 2 of the ranking task*}.

What do these mean to you, and why do you feel so strongly they are the least important to measure from this list?

[*free text box*]

- 1. Is there anything else you would like us to know about your views, or how you made your choices?

[*free text box*]

- Lifeline (24 hours): phone 13 11 14 or [www.lifeline.org.au](http://www.lifeline.org.au/)
- Beyondblue: phone 1300 224 636 or [www.beyondblue.org.au](http://www.beyondblue.org.au/)
- SANE Australia: phone 1800 187 263 or [www.sane.org](http://www.sane.org/)
- Kids Helpline (24 hours): phone 1800 55 1800 or [kidshelpline.com.au](http://www.kidshelpline.com.au/)
- Tandem Carers support and referral line: phone 1800 314 325 or [tandemcarers.org.au](http://www.tandemcarers.org.au/)

☐ Next ***[**proceeds to A little about you questions, below]***

Section 6: Study follow-up

Thank you so much for your time. We really appreciate you sharing your views.

7.1 **Please provide an email address below where we can email your $40 voucher, as a token of our appreciation.**

☐ Email: | [*free text box*]

☐ No thanks, I don’t want the voucher.

7.2 **If you would like to receive a summary of the results of this research via email, please provide an email address below.**

☐ ***[*If an email address is provided to the question above]*** Use same email address as above

☐ Use a different email address: | [*free text box*]

☐ Email: | [*free text box*]

☐ No thanks

7.3 **It is possible that we may be able to conduct further research in the future that is related to this study.** **If you are happy to be contacted about similar research studies in the future, please provide a contact email address below.**

*(Note: Providing your email does not mean you have to participate in the future study, you will still have the choice to not participate after receiving details of any future study).*

☐ ***[*If an email address is provided to one of the questions above]*** Use same email address as above

☐ Use a different email address: | [*free text box*]

☐ Email: | [*free text box*]

☐ No thanks

7.4 **How did you hear about this survey?**

☐ I was emailed by Tandem Carers

☐ I was emailed by the Victorian Mental Illness Awareness Council (VMIAC)

☐ I heard through a family member/supporter/carer who has also completed this survey

☐ Other: |    [free text box]     |

☐ Next ***[**proceeds to Debriefing page, below]***

*****Debriefing Page *****

*****statement for participants aged 15-17 (based on their reported age in survey responses)***

**Please click the SUBMIT button at the bottom of the page to submit your survey responses.**

Before you go!

Completing this survey may have made you to think about your own experiences of mental health challenges and experiences of accessing care.

If this has made you to feel upset or distressed in any way, you should talk to someone you trust – this could include a family member, friend, teacher, school counsellor. You could also seek support from your GP, psychologist or psychiatrist.

It’s important for you to know that distressing thoughts or behaviours might appear days, weeks or even months after completing this survey.

If you start to feel any of these sorts of signs in yourself, we encourage to you seek support:

- Feeling sad or down
- Confused, or lower ability to concentrate
- Worrying a lot or extreme feelings of guilt
- Extreme mood changes – feeling really high and really low
- Withdrawing from friends and activities
- Problems sleeping or feeling more tired than usual
- Problems with alcohol or drug use
- Excessive anger or violence
- Suicidal thinking

**If you need help immediately, please contact emergency services:**

- Visit a hospital emergency department
- Emergency services: phone 000

**There are a wide range of other support services available to help you. Many have confidential online or phone chat lines that are available after hours.**

- Trusted teacher or school counsellor
- Your GP, counsellor, psychologist, or psychiatrist.
- Kids Helpline (24 hours): phone 1800 55 1800 or [www.kidshelpline.com.au](http://www.kidshelpline.com.au)
- Headspace online and phone support: phone 1800 650 890 or [www.headspace.org.au/online-and-phone-support](http://www.headspace.org.au/online-and-phone-support)
- A local Headspace drop-in centre: [Werribee](https://headspace.org.au/headspace-centres/werribee/), [Glenroy](https://headspace.org.au/headspace-centres/glenroy/), [Craigieburn](https://headspace.org.au/headspace-centres/craigieburn/), [Sunshine](https://headspace.org.au/headspace-centres/sunshine/) and [Melton](https://headspace.org.au/headspace-centres/melton/).
- Lifeline (24 hours): phone 13 11 14 or [www.lifeline.org.au](http://www.lifeline.org.au)
- Suicide callback service (24 hours): phone 1300 659 467
- Beyondblue: phone 1300 22 46 36 or [www.beyondblue.org.au](http://www.beyondblue.org.au)
- Tandem Carers support and referral line: phone 1800 314 325 or [tandemcarers.org.au](http://www.tandemcarers.org.au)
- 13 Yarn (24 hours): phone 13 92 76 or [13yarn.org.au](https://www.13yarn.org.au/) – Indigenous-led National Aboriginal and Torres Strait Islander Crisis support line.
- Rainbow Door: phone 1800 729 367, or text 0480 017 246, or email [support@rainbowdoor.org.au](mailto:support@rainbowdoor.org.au) – non-emergency, free specialist LGBTIQA+ helpline providing information, support, and referral to all LGBTIQA+ Victorians, their friends and family.
- Qlife: phone 1800 184 527, or webchat at [qlife.org.au/resources/chat](https://qlife.org.au/resources/chat) - anonymous, LGBTI peer support and referral.
- Carers Victoria: phone 1800 514 845 or [carersvictoria.org.au](https://www.carersvictoria.org.au/)
- Yarning Safe and Strong (24 hours): phone 1800 959 563 or [www.vahs.org.au/yarning-safenstrong](https://www.vahs.org.au/yarning-safenstrong/) – Free and confidential counselling service for Aboriginal and Torres Strait Islander people.
- SANE Australia: phone 1800 18 72 63 or [www.sane.org](http://www.sane.org)

☐ SUBMIT Survey ***[**closes window, submits survey]***

***** SIMILAR DEBRIEFING STATEMENT for participants aged 18+ years (based on their reported age in survey responses)***

**Please click the SUBMIT button at the bottom of the page to submit your survey responses.**

Before you go!

Completing this survey may have caused you to think about your own experiences of mental health challenges and experiences of accessing care.

If this has made you to feel upset or distressed in any way, you should talk to someone you trust – such as a family member or friend. You could also seek support from your GP, psychologist or psychiatrist.

It’s important for you to know that distressing thoughts or behaviours might appear days, weeks or even months after completing this survey.

If you start to feel any of these sorts of signs in yourself, we encourage to you seek support:

- Feeling sad or down
- Confused, or lower ability to concentrate
- Worrying a lot or extreme feelings of guilt
- Extreme mood changes – feeling really high and really low
- Withdrawing from friends and activities
- Problems sleeping or feeling more tired than usual
- Problems with alcohol or drug use
- Excessive anger or violence
- Suicidal thinking

**If you need help immediately, please contact emergency services:**

- Visit a hospital emergency department.
- Emergency services: phone 000

**There are a wide range of other support services available to help you. Many have confidential online or phone chat lines that are available after hours.**

- Your GP, counsellor, psychologist, or psychiatrist.
- Kids Helpline (24 hours, available to parents and carers): phone 1800 55 1800 or [www.kidshelpline.com.au](http://www.kidshelpline.com.au)
- Headspace online and phone support: phone 1800 650 890 or [www.headspace.org.au/online-and-phone-support](http://www.headspace.org.au/online-and-phone-support)
- A local Headspace drop-in centre: [Werribee](https://headspace.org.au/headspace-centres/werribee/), [Glenroy](https://headspace.org.au/headspace-centres/glenroy/), [Craigieburn](https://headspace.org.au/headspace-centres/craigieburn/), [Sunshine](https://headspace.org.au/headspace-centres/sunshine/) and [Melton](https://headspace.org.au/headspace-centres/melton/).
- Lifeline (24 hours): phone 13 11 14 or [www.lifeline.org.au](http://www.lifeline.org.au)
- Suicide callback service (24 hours): phone 1300 659 467
- Beyondblue: phone 1300 22 46 36 or [www.beyondblue.org.au](http://www.beyondblue.org.au)
- Tandem Carers support and referral line: phone 1800 314 325 or [tandemcarers.org.au](http://www.tandemcarers.org.au)
- 13 Yarn (24 hours): phone 13 92 76 or [13yarn.org.au](https://www.13yarn.org.au/) – Indigenous-led National Aboriginal and Torres Strait Islander Crisis support line.
- Rainbow Door: phone 1800 729 367, or text 0480 017 246, or email [support@rainbowdoor.org.au](mailto:support@rainbowdoor.org.au) – non-emergency, free specialist LGBTIQA+ helpline providing information, support, and referral to all LGBTIQA+ Victorians, their friends and family.
- Qlife: phone 1800 184 527, or webchat at [qlife.org.au/resources/chat](https://qlife.org.au/resources/chat) - anonymous, LGBTI peer support and referral.
- Carers Victoria: phone 1800 514 845 or [carersvictoria.org.au](https://www.carersvictoria.org.au/)
- Yarning Safe and Strong (24 hours): phone 1800 959 563 or [www.vahs.org.au/yarning-safenstrong](https://www.vahs.org.au/yarning-safenstrong/) – Free and confidential counselling service for Aboriginal and Torres Strait Islander people.
- SANE Australia: phone 1800 18 72 63 or [www.sane.org](http://www.sane.org)
- Eastern Access Community Health (EACH): phone 1300 003 224 or [www.each.com.au](http://www.each.com.au)
- MensLine Australia: phone 1300 78 99 78 or [www.mensline.org.au](http://www.mensline.org.au)

☐ SUBMIT Survey ***[**closes window, submits survey]***

**[ END ONLINE SURVEY ]**

# **Supplementary Material 2.** The Q-sort distribution grid.

**Figure S2.1.** The Q-sort distribution grid used in analyses.


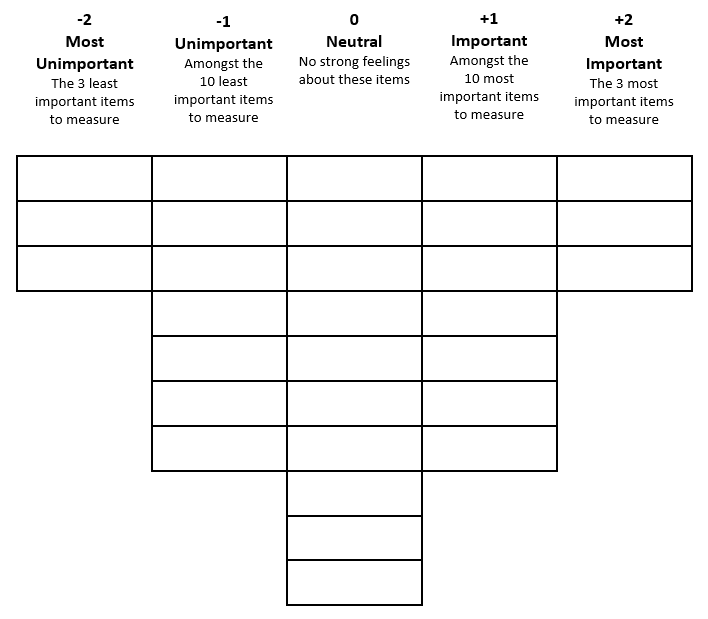


# **Supplementary Material 3.** Output from Principal component analysis: Determining the number of factors to be extracted.

1. Unrotated principal components and eigenvalues


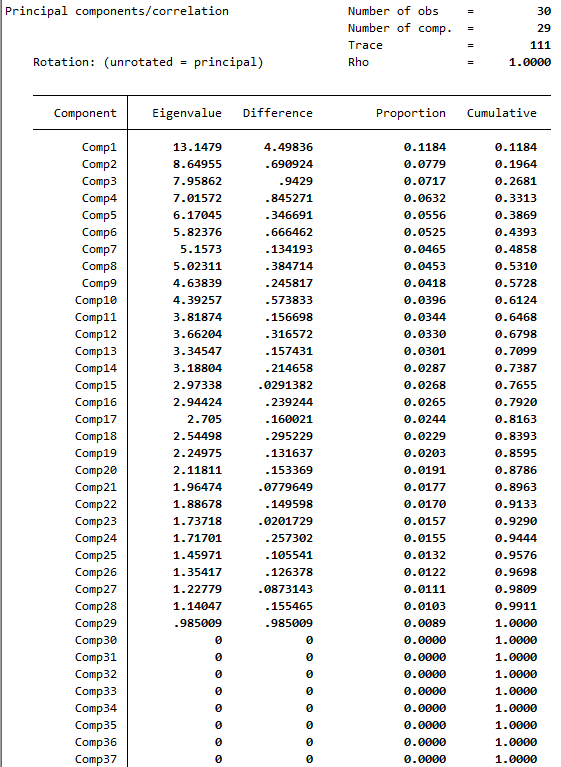


Result: Eigenvalues > 1.0 suggests 28 components to be retained

1. Scree plot

Result: Scree plot is ambiguous. No clear number of components to retain.

1. Horn’s Parallel Analysis


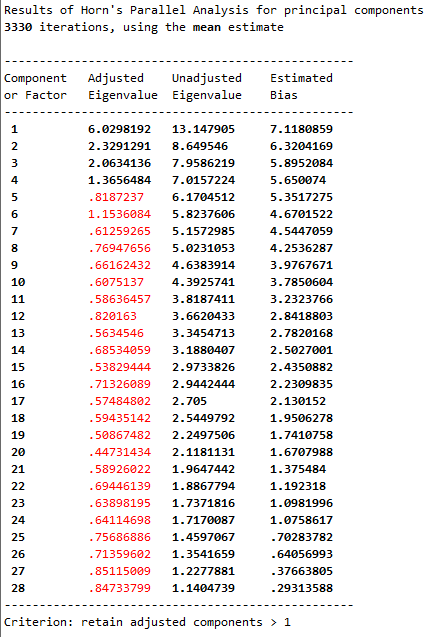

Decision: Results of the Kaiser-Guttman and Scree plot tests indicated 28 factors should be retained; whereas Horn’s Parallel Analysis indicated 4 factors were appropriate to retain. As these tests were not in agreeance, and the Parallel Analysis is considered more accurate than the Kaiser or Scree methods [1], we focused our analyses on the 4 most common factors (“viewpoints”). retain 4 components for interpretation.

[1] Schmitt TA. Current methodological considerations in exploratory and confirmatory factor analysis. J Psychoeduc Assess. 2011;29(4):304–21.

1. **Rotated factor scores**

Rotation technique = orthogonal varimax

Method = principal component factors

**Communality** shows the proportion of each participants’ variability that is explained by the four factors; i.e. how well each participant’s viewpoint is captured by these factors.

**Variance** shows the proportion of variation in the data explained by each factor; i.e. how well each viewpoint represents the range of views that exist in the data.

**Bold** factor scores are scores > 0.30, considered a moderate correlation[3] with the factor.

**Bold shaded** factor scores show how each participant was loaded onto the main four factors.

| **Participant** | **Factor1** | **Factor2** | **Factor3** | **Factor4** | **Communality** |
| --- | --- | --- | --- | --- | --- |
| v1 | 0.040 | 0.084 | -0.055 | **-0.328** | 0.120 |
| v2 | 0.109 | 0.222 | -0.211 | **0.316** | 0.205 |
| v3 | **0.667** | 0.194 | -0.010 | 0.195 | 0.520 |
| v4 | -0.143 | -0.181 | 0.099 | **-0.531** | 0.345 |
| v5 | 0.284 | 0.070 | 0.150 | **-0.416** | 0.281 |
| v6 | **0.554** | 0.053 | -0.022 | -0.019 | 0.311 |
| v7 | **0.596** | -0.181 | 0.181 | -0.143 | 0.441 |
| v8 | **-0.644** | 0.195 | -0.107 | 0.005 | 0.464 |
| v9 | -0.066 | **-0.418** | -0.193 | 0.190 | 0.252 |
| v10 | 0.148 | **-0.534** | -0.012 | 0.222 | 0.357 |
| v11 | 0.014 | **0.442** | -0.175 | **-0.322** | 0.329 |
| v12 | 0.283 | -0.017 | -0.201 | -0.172 | 0.151 |
| v13 | -0.076 | 0.043 | **-0.432** | -0.054 | 0.197 |
| v14 | 0.087 | 0.167 | 0.059 | **-0.512** | 0.301 |
| v15 | **0.591** | 0.255 | 0.019 | 0.297 | 0.502 |
| v16 | **0.546** | 0.028 | **0.540** | 0.006 | 0.591 |
| v17 | 0.296 | -0.185 | -0.397 | **0.596** | 0.635 |
| v18 | -0.072 | 0.108 | 0.176 | **0.593** | 0.399 |
| v19 | -0.057 | 0.081 | -0.035 | **0.413** | 0.182 |
| v20 | 0.120 | 0.197 | 0.075 | 0.160 | 0.084 |
| v21 | 0.130 | 0.016 | -0.273 | **0.472** | 0.315 |
| v22 | 0.068 | 0.098 | -0.252 | **0.503** | 0.330 |
| v23 | -0.107 | 0.051 | **0.304** | **0.352** | 0.230 |
| v24 | 0.300 | **0.554** | -0.154 | 0.026 | 0.422 |
| v25 | 0.016 | -0.083 | -0.200 | 0.134 | 0.065 |
| v26 | 0.074 | 0.297 | -0.074 | **0.501** | 0.350 |
| v27 | 0.042 | **0.394** | 0.038 | 0.114 | 0.171 |
| v28 | -0.157 | **0.677** | **0.398** | 0.009 | 0.641 |
| v29 | 0.081 | 0.050 | **0.365** | **-0.427** | 0.324 |
| v30 | **0.580** | 0.285 | -0.001 | 0.291 | 0.502 |
| v31 | 0.081 | 0.087 | 0.129 | -0.091 | 0.039 |
| v32 | **0.516** | **0.349** | 0.029 | -0.195 | 0.427 |
| v33 | 0.260 | **0.414** | -0.206 | 0.176 | 0.313 |
| v34 | **0.484** | -0.032 | -0.073 | **0.441** | 0.435 |
| v35 | 0.074 | 0.063 | 0.120 | **0.357** | 0.152 |
| v36 | 0.172 | **-0.354** | **0.336** | 0.180 | 0.301 |
| v37 | 0.225 | **0.412** | 0.006 | 0.001 | 0.220 |
| v38 | -0.120 | -0.145 | **0.601** | -0.027 | 0.397 |
| v39 | 0.027 | **0.401** | **0.543** | 0.104 | 0.468 |
| v40 | **0.453** | 0.167 | 0.030 | -0.017 | 0.234 |
| v41 | **0.391** | **0.422** | **0.394** | -0.216 | 0.532 |
| v42 | **0.366** | 0.127 | -0.268 | 0.003 | 0.222 |
| v43 | -0.087 | **-0.502** | -0.116 | -0.077 | 0.279 |
| v44 | -0.067 | 0.017 | 0.039 | 0.065 | 0.011 |
| v45 | **0.514** | 0.002 | 0.154 | -0.224 | 0.337 |
| v46 | 0.117 | **-0.367** | -0.081 | 0.187 | 0.190 |
| v47 | 0.081 | -0.074 | -0.041 | **0.713** | 0.522 |
| v48 | 0.009 | 0.114 | 0.132 | **0.645** | 0.446 |
| v49 | **-0.327** | 0.035 | **0.369** | **-0.359** | 0.373 |
| v50 | -0.076 | -0.079 | **0.353** | **0.694** | 0.618 |
| v51 | 0.276 | 0.217 | 0.005 | 0.107 | 0.135 |
| v52 | 0.009 | **0.364** | 0.026 | 0.040 | 0.135 |
| v53 | **0.460** | 0.091 | 0.219 | 0.235 | 0.323 |
| v54 | -0.038 | -0.051 | 0.166 | 0.095 | 0.041 |
| v55 | 0.124 | 0.258 | -0.080 | **0.336** | 0.201 |
| v56 | 0.005 | 0.082 | **-0.372** | -0.071 | 0.150 |
| v57 | 0.273 | 0.034 | **-0.424** | 0.031 | 0.256 |
| v58 | -0.035 | 0.174 | **0.597** | -0.035 | 0.389 |
| v59 | **-0.343** | 0.288 | 0.018 | 0.057 | 0.204 |
| v60 | 0.247 | -0.055 | **0.380** | 0.022 | 0.209 |
| v61 | **0.358** | 0.243 | **0.549** | -0.030 | 0.489 |
| v62 | 0.054 | **0.347** | 0.219 | 0.242 | 0.230 |
| v63 | **0.743** | **0.366** | 0.064 | -0.084 | 0.697 |
| v64 | **0.656** | -0.073 | -0.079 | -0.075 | 0.448 |
| v65 | **0.371** | -0.092 | -0.041 | 0.148 | 0.170 |
| v66 | -0.089 | 0.129 | **0.350** | 0.099 | 0.157 |
| v67 | 0.016 | -0.206 | 0.015 | -0.026 | 0.044 |
| v68 | **-0.309** | -0.006 | 0.178 | **0.684** | 0.595 |
| v69 | 0.085 | **0.310** | **0.627** | 0.042 | 0.499 |
| v70 | 0.050 | **0.407** | 0.066 | **0.382** | 0.318 |
| v71 | -0.138 | -0.227 | 0.170 | 0.085 | 0.107 |
| v72 | -0.049 | -**0.349** | -0.103 | 0.244 | 0.194 |
| v73 | 0.123 | 0.222 | **0.490** | -0.005 | 0.304 |
| v74 | **0.402** | **0.450** | **0.304** | 0.180 | 0.489 |
| v75 | **0.542** | 0.082 | **0.370** | -0.092 | 0.446 |
| v76 | 0.211 | 0.273 | 0.142 | 0.103 | 0.150 |
| v77 | -0.036 | **0.445** | **0.382** | -0.183 | 0.378 |
| v78 | -0.043 | **-0.330** | 0.298 | **0.343** | 0.317 |
| v79 | 0.022 | 0.005 | 0.196 | **0.350** | 0.161 |
| v80 | 0.096 | **0.480** | -0.123 | 0.224 | 0.305 |
| v81 | **0.545** | 0.047 | -0.162 | 0.146 | 0.347 |
| v82 | **0.585** | -0.076 | 0.024 | 0.243 | **0.407** |
| v83 | 0.028 | **0.554** | **0.321** | -0.226 | **0.462** |
| v84 | **0.320** | 0.052 | 0.086 | -0.157 | 0.137 |
| v85 | **0.460** | 0.261 | -0.142 | -0.270 | 0.373 |
| v86 | **0.441** | 0.195 | -0.282 | 0.113 | 0.325 |
| v87 | 0.017 | **0.614** | **-0.309** | 0.133 | 0.490 |
| v88 | 0.196 | 0.119 | **0.686** | **-0.357** | 0.650 |
| v89 | **0.311** | **-0.494** | 0.170 | 0.285 | 0.450 |
| v90 | **0.346** | 0.086 | **0.482** | 0.233 | 0.414 |
| v91 | 0.055 | -0.259 | -0.208 | 0.273 | 0.188 |
| v92 | **0.386** | 0.238 | 0.069 | -0.307 | 0.305 |
| v93 | **0.445** | -0.243 | **0.549** | 0.100 | 0.568 |
| v94 | **0.349** | **0.391** | -0.065 | 0.216 | 0.325 |
| v95 | **0.520** | **0.463** | 0.283 | -0.039 | 0.566 |
| v96 | **0.680** | -0.276 | 0.206 | -0.218 | 0.628 |
| v97 | -0.074 | -0.203 | 0.123 | **0.420** | 0.238 |
| v98 | 0.202 | 0.211 | -**0.378** | 0.160 | 0.254 |
| v99 | 0.080 | **0.614** | -0.045 | 0.198 | 0.425 |
| v100 | 0.275 | 0.269 | 0.087 | -0.204 | 0.197 |
| v101 | **-0.495** | 0.160 | -0.243 | 0.108 | 0.342 |
| v102 | -0.029 | **0.688** | -0.132 | 0.175 | 0.522 |
| v103 | 0.018 | **0.504** | 0.201 | -0.150 | 0.317 |
| v104 | **-0.328** | **0.487** | -0.145 | **0.419** | 0.541 |
| v105 | 0.167 | **0.374** | 0.069 | -0.042 | 0.174 |
| v106 | 0.141 | -0.125 | **0.646** | 0.130 | 0.470 |
| v107 | 0.036 | **0.516** | **0.354** | -0.033 | 0.394 |
| v108 | 0.234 | -0.254 | -0.208 | -0.054 | 0.166 |
| v109 | 0.267 | **0.363** | **0.386** | **0.395** | 0.508 |
| v110 | -0.206 | **0.310** | -0.091 | -0.014 | 0.147 |
| v111 | 0.297 | 0.108 | **0.517** | -0.081 | 0.374 |
| **VARIANCE** | **10.455** | **9.587** | **8.397** | **8.331** |  |

# **Supplementary Material 4.** Results of subgroup analysis and crosstabs for consumers and carers and each age group separately.

The below tables show the overlap of common viewpoints that emerge from the main analysis in the combined sample (4 viewpoints); overlapping with viewpoints that emerge from the consumer-only analysis (2 viewpoints); carer-only analysis (6 viewpoints); carer+consumer analysis (2 viewpoints); 15-24 year old analysis (2 viewpoints); 25-64 year old analysis (4 viewpoints); and 65+ year old analysis (3 viewpoints).

1. **Consumer views:**


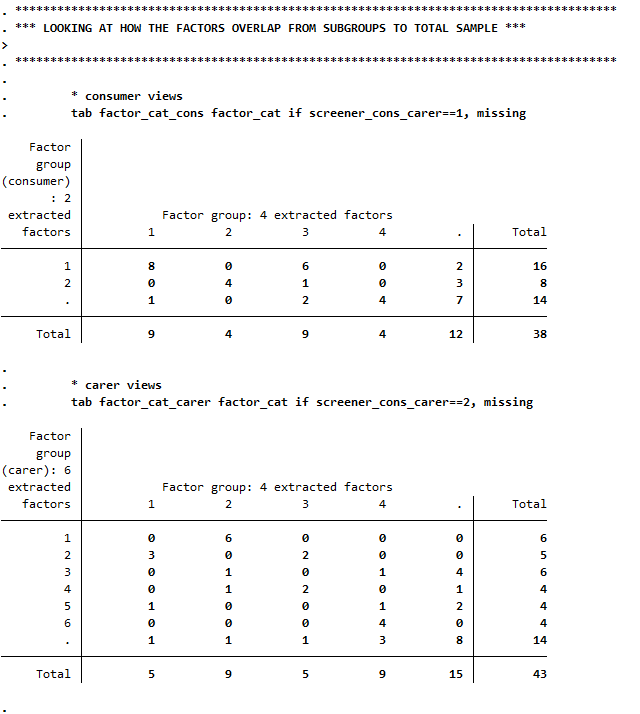


Interpretation, consumers:

- Consumer Viewpoint #1 represents a combination of the main viewpoints #1 and #3.
- Consumer viewpoint #2 is reflected in main viewpoint #2.
- Main viewpoint #4 was not a viewpoint commonly held by consumers.

1. **Carer views:**


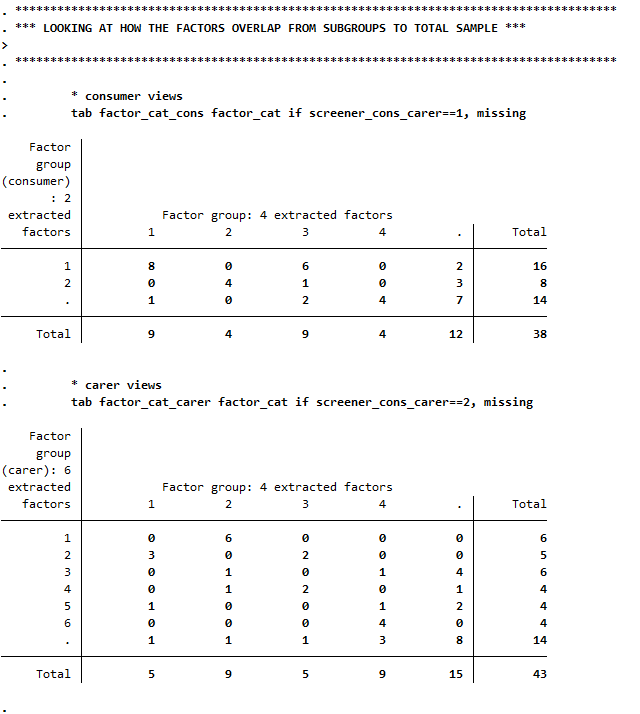


Interpretation, carers:

- Carer viewpoint #1 was accurately reflected by main viewpoint #2
- Carer viewpoints #2 represents a combination of main viewpoint #1 and #3
- Carer viewpoint #3 was largely not reflected by the main viewpoints, but may include aspects of main view #2 and #4.
- Carer viewpoint #4 was mostly represented by main view #3
- Carer viewpoint #5 included aspects of main view #1 and #4.
- Carer viewpoint #6 was accurately reflected in main view #4.

1. **Carer+consumer views:**


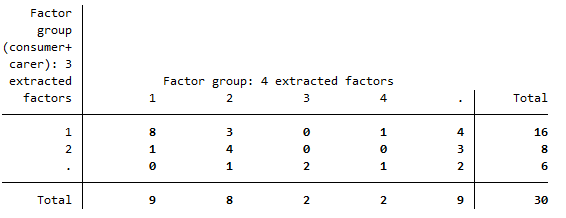


Interpretation, carer+consumers:

- Carer+consumer viewpoint #1 was largely reflected in Main Viewpoint #1.
- Carer+consumer viewpoint #2 was largely reflected in Main Viewpoint #2.

1. **15-24 year olds**


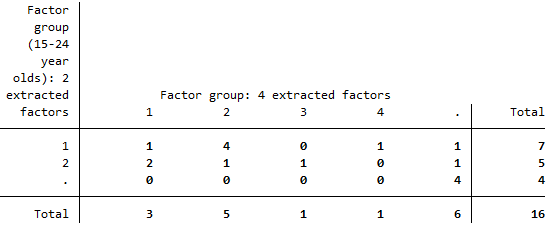


Interpretation, 15-24 year olds:

- The two common viewpoints of 15-24 year olds were captured as min viewpoints #1 and #2
- Further examination showed three 15-17 year olds were in the ‘missing’ categories here – their views were not captured by the common viewpoints in either the X or Y axis.
- This means the common viewpoints here are more reflective of 18-24 year olds (rather than 15-24 year olds)

1. **25-64 year olds**


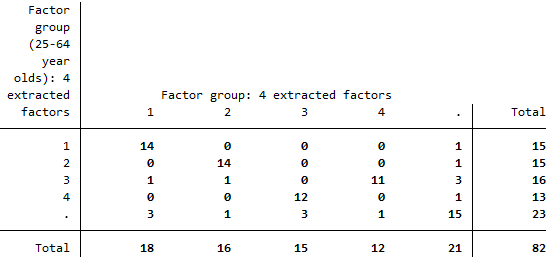


Interpretation, 25-64 year olds:

- 4 Viewpoints of 25-64 year olds almost perfectly overlapped with the 4 main viewpoints

1. **65+ year olds**


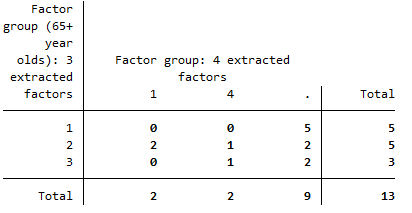


Interpretation, 65+ year olds:

- Most common view of 65+ year olds was not one of the 4 main viewpoints
- Second most common view of 65+ year olds was Viewpoint #1.
